# Calm beats cute: designing aesthetic-functional congruence in human-robot interaction for breathing companion robots

**DOI:** 10.3389/fcogn.2026.1886795

**Published:** 2026-09-23

**Authors:** Ilona Buchem, Rinat B. Rosenberg-Kima, Emily Evermann, Mandana Sutter

**Affiliations:** 1 Communication Laboratory, Department of Economics and Social Sciences, Berlin University of Applied Sciences, Berlin, Germany; 2 The Mindful Learning Technologies Lab, Faculty of Education in Science and Technology, Technion – Israel Institute of Technology, Haifa, Israel; 3 Faculty of Mechanical Engineering and Transport Systems Faculty, Institute of Psychology and Ergonomics, Technical University of Berlin, Berlin, Germany

**Keywords:** aesthetics, breathing companion robot, embodiment, human-likeness, human–robot interaction, social robots, technology acceptance, user experience

## Abstract

**Introduction:**

Which robot would you trust to guide you through a breathing exercise, a cute cartoon-like humanoid, a soft animal companion, a clinical mechanical device, or a serene meditative figure? This study examines how four design aesthetics affect social evaluation and technology acceptance of Neffy, a social breathing-companion robot that supports stress relief and wellbeing by guiding exercises through visual, audio, and haptic cues synchronized with upper-body movement.

**Methods:**

In an online study, 126 participants viewed a video of a person interacting with Neffy during a guided breathing exercise. Four design styles were tested: cartoonish humanoid, meditative humanoid, animal-like (zoomorphic), and machine-like (mechanomorphic). Participants rated each style on warmth, competence, perceived enjoyment, and intention to use, and made pairwise comparisons of human-likeness, social presence, trustworthiness, breathing companion preference, and overall liking.

**Results:**

The zoomorphic design received the highest warmth ratings, the mechanomorphic design scored lowest across all measures. Perceived human-likeness positively predicted both warmth and competence, confirming its role in social evaluation. However, human-likeness alone did not determine preference: despite comparable perceived human-likeness between the two humanoid designs, the meditative humanoid achieved the highest scores for competence, trustworthiness, and breathing companion preference, outperforming the cartoon-like humanoid.

**Discussion:**

These results suggest that “calm” aesthetics are preferred over “cute” aesthetics for wellbeing robots, challenging the assumption that human-likeness alone drives design success. Instead, aesthetic-functional congruence-alignment between a robot's visual design and its functional purpose, appears to be a critical, under-explored factor in social robot design for wellbeing. Since this study assessed general evaluations, future work should test whether these preferences hold across application domains (e.g. clinical, workplace, educational, domestic) and cross-cultural contexts.

## 1 Introduction

The increasing integration of social robots into education, healthcare, and wellbeing raises a fundamental design question: What should a robot look like in order to be accepted, trusted, and willingly used? Visual design is typically the first and most salient cue users encounter, shaping expectations about a robot's capabilities, intentions and social roles before any interaction takes place (Fong et al., 2003; Syrdal et al., 2007). From the perspective of design aesthetics, visual design of a robot acts as a communication system through which a robot conveys its affordances, symbolism and meaning, and guides users' evaluation, understanding and interaction (Norman, 2013; Mobed et al., 2024). While technical functionality is an essential prerequisite for effective robotic systems, it alone is insufficient to guarantee positive user experiences or sustained use (Colle and Dragone, 2026). A coherent alignment of visual design, embodied behavior, and symbolic framing, which functions as “product semantics” (Mobed et al., 2024), is especially relevant for companion robots intended for personal wellbeing (Colle and Dragone, 2026).

Robot morphology has been considered as a fundamental characteristic of robot design. It encompasses the categorization of a robot's physical form and represents a key element of robot aesthetics, conveying the robot's intended purpose and functionality (Mobed et al., 2024). Despite ample research on robot morphology, much of the research has focused on the examination of isolated visual features in robots, not sufficiently accounting for the multidimensional character of the aesthetic experience (Colle and Dragone, 2026). While no consensus has emerged so far on what constitutes effective robot design, an increasing body of research suggests that robot design should be understood as a multidimensional construct in which multiple design characteristics including morphology, materials, colors, shapes, movements, and affective expression jointly influence social perceptions of robots, e.g., in relation to perceived warmth and competence (Colle and Dragone, 2026). It has been argued that robot appeal to a large extent may be influenced by the coherence between how the robot looks like and its behavior (Colle and Dragone, 2026). In this paper we define aesthetic-functional congruence in robot design as the alignment between a robot's visual design and its functional purpose. Following Colle and Dragone (2026), we argue that a congruence between technical functionality and aesthetic design may be reached when both are designed as a coherent whole. As the relationship between appearance and acceptance is not universal and not reducible to a single dimension such as morphology or human-likeness, the present study examines the relationship between different types of design aesthetics in the context of a breathing companion robot, in which aesthetic-functional congruence may matter more than morphological design categories or human-likeness alone. Building on the work by Colle and Dragone (2026), we view aesthetics in robot design as “a constructed experience” in which users integrate perceptual cues of a robot with their symbolic framings which results from individual preferences, experiences, prior knowledge, social and cultural contexts. Viewing aesthetics as a mechanism for design (Mobed et al., 2024) shifts the perspective from aesthetics as a “nice-to-have” aspect of “surface styling” to a fundamental design mechanism that shapes users' aesthetic responses (Colle and Dragone, 2026).

### 1.1 From appearance to social perception: the role of anthropomorphism

The appearance of a robot is usually the first point of contact. The sensory cues of a robot affect human interpretations, e.g., a cartoon style robot design can create an emotional connection and build trust, familiar zoomorphic designs can foster bonding, while anthropomorphic designs can result in inappropriate social expectations (Mobed et al., 2024). A foundational insight from human-robot interaction (HRI) research is that humans readily apply social interpretations to artificial agents, even those with minimal social features. The Computers Are Social Actors (CASA) paradigm explains this as an automatic and unconscious activation of social-cognitive processes triggered by perceptual cues such as faces, voices, or behaviors, which do not require conscious belief that the system is sentient (Nass et al., 1994; Heyselaar, 2023). While CASA theory describes unconscious, social reactions to computers, anthropomorphism refers to the conscious attribution of human characteristics, intentions, and emotions to non-human entities (Heyselaar, 2023). Although anthropomorphic design cues may strengthen social responses, the CASA framework suggests that social reactions occur automatically and do not depend on users explicitly perceiving a robot as human-like (Heyselaar, 2023). Importantly, anthropomorphism, i.e., the attribution of human-like characteristics, intentions, and emotions to non-human entities (Nass et al., 1994; Mergner et al., 2019), is not a binary property but a multidimensional, graded phenomenon that can operate through appearance, behavior, or functional performance together or independently (Bhatti et al., 2026). For example, a robot may move in human-like ways without looking human, or display a human face without behaving in human-like ways. This distinction is relevant for breathing companion robots, which simulate a human physiological process, i.e., the rhythmic rise and fall of the upper torso simulating inhalation and exhalation, without necessarily adopting a human visual form. Breathing companion robots, such as Neffy, represent a case of functional anthropomorphism without strong material anthropomorphism (Mergner et al., 2019) as its functional meaning is grounded in sensorimotor simulation of the torso rather than visual resemblance to breathing humans or animals.

### 1.2 The design space: strategies to design robot appearance

Beyond humanoid design, social robots may have zoomorphic, mechanomorphic, or symbolic morphologies, each conveying distinct perceptual and social cues (Fong et al., 2003; Roesler et al., 2023). Empirical findings consistently show that these different morphological strategies produce distinct patterns of user evaluation, and that human-like design is not universally optimal. For example, zoomorphic designs, especially those resembling familiar, soft animals, have been associated with higher perceived warmth, emotional approachability, and user acceptance, especially among children (Wróbel et al., 2024) and older adults (Shibata and Wada, 2011; Schnitzer et al., 2024; Xiao et al., 2025). In therapeutic contexts, soft materials and tactile interactions afforded by zoomorphic robots have been shown to provide practical benefits for emotional connection and functional outcomes (Šabanović et al., 2013; Matheus et al., 2025). By contrast, mechanomorphic designs, which may be appropriate for task-oriented or industrial contexts, tend to be rated lower on relational and affective dimensions in other application contexts (Pohlt et al., 2018; Weiss and Huber, 2016). Studies have shown that anthropomorphic designs can enhance likeability and perceived social presence of robots, particularly when paired with appropriate interaction modalities such as human-like voice (Klüber Onnasch and Onnasch, 2022). However, such designs carry the risk of raising user expectations beyond the robot's actual capabilities (Bradwell et al., 2021). This risk is most acute at high levels of human realism, as captured by Mori's (1970) Uncanny Valley hypothesis, which proposes that evaluations of robots become increasingly positive as human-likeness increases until a threshold of near-realism is crossed, at which point familiarity changes into unease and rejection. Importantly, the Uncanny Valley implies that moderate human-likeness, achieved through simplified or stylized rather than realistic faces, may represent a sweet spot for social robot design. Research on facial design has shown that round eyes, upturned or neutral mouths, and baby schema proportions, i.e., features associated with cuteness and safety, tend to be perceived as non-threatening and produce more positive evaluations than either purely abstract or highly realistic face designs (Blow et al., 2006; Kalegina et al., 2018; Chen and Jia, 2023; Song and Luximon, 2024). However, preferences for robot appearance are heterogeneous, context- and function-dependent, and no single design strategy has been shown to be universally effective (cf. Kim et al., 2021; Woods, 2006; Robins et al., 2004; Rosenberg-Kima and Buchem, 2025).

### 1.3 Toward a framework of aesthetic-functional congruence in HRI

To move beyond the question of which morphological category is best for eliciting users' positive responses, e.g., in terms of social evaluations and technology acceptance, the present study proposes that robot design effectiveness in wellbeing contexts may be better understood through the concept of aesthetic-functional congruence, i.e., the degree to which a robot's visual and emotional aesthetic design aligns with the functional purpose and the context of its intended use. Aesthetic experiences refer to an experience in which a sensory entity triggers a process of evaluation of a particular concept such as “beauty” (Jacobsen, 2006). At the cognitive level, the aesthetic mode of processing is characterized by an attentional focus on the stimulus during an integration of context, memory and sensory qualities of the stimulus (Weigand and Jacobsen, 2025). The proposed framework of aesthetic-functional congruence integrates insights from three theoretical perspectives.

First, the Affordance Theory, describes affordances as action possibilities that emerge from the relationship between an object and a user, rather than being fixed properties of the object itself (Gibson, 1979; Norman, 2013). In HRI, robot embodiment functions as a carrier of affordances, e.g., visual and physical features signal what kind of interaction is possible and appropriate. The Form-Affordance-Function (FAF) framework formalizes this by treating visual form, affordances, and function as mutually constraining, i.e., optimizing one element (e.g., aesthetic appearance) may reduce the clarity of another (e.g., the affordance of placing hands on the robot's shoulders for breathing synchronization; Sareh and Loudon, 2024). Also, affordances are distinguished from signifiers, i.e., while affordances, such as shape of the body, define possible interactions, signifiers, such as light and voice, communicate how those interactions should be performed (Norman, 2013). Aesthetic–functional congruence means that the robot's visual design should be congruent with the signifiers and affordances of the intended use of the robot, e.g., slow-paced breathing.

The second perspective of the Schema Congruity Theory proposes that individuals evaluate objects based on their fit with pre-existing cognitive schemas, i.e., organized knowledge structures that guide perception and interpretation (Meyers-Levy and Tybout, 1989; Campbell and Goodstein, 2001; Madhavan and Wiegmann, 2007; Lou et al., 2022; Gao et al., 2022). The relationship between schema congruity and evaluation follows an inverted U-shape, such that perfectly congruent stimuli may be processed fluently but perceived as boring, while incongruent stimuli may be too confusing and eventually rejected. An optimal solution can be achieved with moderately incongruent stimuli, i.e., those that slightly deviate from expectations but can be meaningfully interpreted (Meyers-Levy and Tybout, 1989; Campbell and Goodstein, 2001). Such moderately incongruent stimuli often lead to more favorable evaluations because the interpretable novelty stimulates cognitive elaboration, curiosity and deeper processing (Meyers-Levy and Tybout, 1989; Gao et al., 2022). Applied to breathing companion robots, users likely hold two competing schemas simultaneously, i.e., a robot schema (robot as a functional, task-oriented, technological object) and a wellbeing schema of slow breathing exercises (robot as an aesthetically appealing...companion and supportive companion enhancing sensory experience rather than a technology merely fulfilling functional tasks). A robot design that activates only the robot schema may feel inappropriate for slow-paced breathing exercises aimed at stress reduction. At the same time, a robot that activates only the wellbeing schema may not be recognized as a functional or reliable device. From this perspective, aesthetic-functional congruence in design means aiming to attain a moderate incongruity level between these two possible schemas in a way that is potentially resolvable, i.e., surprising but not confusing (Meyers-Levy and Tybout, 1989; Campbell and Goodstein, 2001). Prior research suggests that the perceived appropriateness and acceptance of robots depend on the congruence between a robot's appearance, behavior, and intended task. For example, Goetz et al. (2003) demonstrated empirically that users preferred robots whose level of human-likeness matched the sociability required by the task and showed greater cooperation with robots whose behavior aligned with task seriousness (Goetz et al., 2003). These findings indicate that users interpret robots through task-related cognitive and social schemas, using appearance and behavior as cues for expected capabilities and roles. Applied to Neffy, this perspective suggests that different visual designs may vary in their perceived fit with the robot's function for slow-paced breathing support. Since individuals may hold fundamentally different schemas about robots, these schemas may in turn influence their judgment of robots' competence and warmth (Lou et al., 2022).

Therefore, the third perspective in the proposed aesthetic-functional congruence framework is the Stereotype Content Model (SCM), which organizes social judgments along two fundamental dimensions: (a) warmth, which reflects perceived friendliness, trustworthiness, and positive intentions, and (b) competence, which reflects perceived ability, intelligence, and effectiveness (Sevillano and Fiske, 2016). Warmth and competence are two complementary dimensions applied to the evaluation of social robots and provide a useful framework for distinguishing relational and functional aspects of robot perceptions (Sevillano and Fiske, 2016). Research applying the SCM to HRI shows that warmth and competence perceptions are shaped by different aspects of robot design. For example, warmth tends to be influenced by appearance and interaction style, while competence tends to be more sensitive to behavior and task performance (Bergmann et al., 2012). These findings indicate that perceived warmth may depend more on robot's appearance, while perceived competence may depend more on robot's behavior. Huang and Moore (2023) found that users expected higher competence than warmth from social robots across multiple roles, and that more human-like appearances were associated with warmth but not with competence. This dissociation is directly relevant to aesthetic–functional congruence, as a design that communicates emotional and relational qualities of warmth while simultaneously signaling functional reliability of competence may represent an optimal balance for wellbeing social robots. However, it remains a research question of how different embodiments may differentially communicate warmth and competence to different users.

Together, these three frameworks converge on a shared outcome being that the most effective design of appearance of a social robot supporting slow-paced breathing is not necessarily the most human-like one, but rather the one whose aesthetic design most coherently communicates a combination of functional purpose and relational qualities of a social robot. Aesthetic-functional congruence in this study refers to the alignment between a robot's visual design and the functional qualities of the intended interaction. This concept is different from matching of human-like morphologies with human-like tasks as it refers to schema activation rather than morphological resemblance.

### 1.4 User experience and technology acceptance

Beyond social evaluations, research on human-technology interaction commonly conceptualizes user experience (UX) as comprising two complementary dimensions: pragmatic and hedonic attributes (Hassenzahl and Tractinsky, 2006; Hassenzahl, 2018). Pragmatic attributes are related to the degree to which a system supports goal achievement effectively and efficiently, while hedonic attributes are related to the experiential and emotional qualities of interaction (Hassenzahl and Tractinsky, 2006; Hassenzahl, 2018). Both pragmatic and hedonic attributes are essential for shaping user experience of interactive systems (Hassenzahl and Tractinsky, 2006). While pragmatic qualities ensure task completion, hedonic qualities influence users' emotional responses, motivation, and long-term engagement. These concepts can be transferred to HRI as pragmatic attributes are associated with the robot's perceived usefulness, usability, and effectiveness in supporting task execution, whereas hedonic attributes relate to users' affective responses, such as liking, perceived enjoyment and pleasantness of the interaction (Pohlt et al., 2018; Weiss and Huber, 2016). Prior work suggests that in settings such as industrial human-robot collaboration, pragmatic attributes tend to be prioritized as factors critical for task performance (Pohlt et al., 2018; Weiss and Huber, 2016), while in domains involving close social interactions, such as care and wellbeing, hedonic attributes become more important, as they contribute to the feelings of perceived companionship and engagement (Chatzoglou et al., 2024).

The Technology Acceptance Model (TAM) offers a well-established framework for understanding users' intentions to use a technology and operationalizes acceptance though a range of factors including perceived usefulness, ease of use, and perceived enjoyment as predictors of behavioral intention to use a technology (Davis, 1989; Venkatesh and Davis, 2000; Venkatesh et al., 2003). In contexts without direct interaction experience, such as the present image-based study, perceived enjoyment and behavioral intention serve as the most relevant indicators of anticipated acceptance. The Partner Choice Framework adds a complementary perspective, suggesting that users may also evaluate robots as potential interaction partners based on perceived trustworthiness and expected relational benefits (Campennì and Schino, 2014). However, research examining the extent to which preferences for specific robot appearance designs reflect such partner-selection processes remains scarce in HRI. Despite extensive research on human-likeness and embodiment of social robots, there is still limited understanding of how different aesthetic designs influence social evaluations and acceptance, particularly in relation to social robots designed to support human wellbeing.

### 1.5 The present study

The question of how different robot aesthetic design styles influence user evaluations in wellbeing contexts remains under-explored. Existing work typically examines isolated facial features or single embodiment categories, without systematically comparing how different aesthetic concepts communicate functional and relational meaning (Colle and Dragone, 2026). Recent work has begun to investigate the design of breathing companion robots. For example, Matheus et al. (2025) presented the design and development of Ommie, a social robot for anxiety reduction, demonstrating the growing interest in embodied robots supporting wellbeing. Inspired by the Ommi robot, a breathing companion robot Neffy was presented by Buchem et al. (2026b). However, both previous contributions primarily focused on the development and evaluation of a single robot design rather than systematically comparing alternative appearance concepts. The role of aesthetic-functional congruence has not been previously directly investigated in the context of breathing support robots. To the best of our knowledge, no previous study has systematically compared multiple aesthetic designs for a breathing companion robot to examine how such designs influence social evaluations of the robot.

The present study addresses this gap through an online study with 126 participants who evaluated visual stimuli representing four visual design variants of the breathing companion robot Neffy. The study builds on the proposed aesthetic-functional congruence framework, which combines the Affordance Theory (Gibson, 1979; Norman, 2013), Form-Affordance-Function (FAF) framework (Sareh and Loudon, 2024), Schema Congruity Theory (Meyers-Levy and Tybout, 1989; Campbell and Goodstein, 2001), and the Stereotype Content Model (SCM; Sevillano and Fiske, 2016), the pragmatic-hedonic distinction (Hassenzahl and Tractinsky, 2006), and the Partner Choice Framework (Campennì and Schino, 2014). The following research questions guided the study:

**RQ1:** How do different robot design styles influence users' perceptions of human-likeness, warmth, competence, perceived enjoyment, and intention to use?
**RQ2:** How do users comparatively evaluate different design styles across social perception dimensions including social presence, trustworthiness, human-likeness, and pragmatic and hedonic preferences?
**RQ3:** How does perceived human-likeness relate to social evaluation, technology acceptance, social presence, trustworthiness, enjoyment, and behavioral intention across design styles?

Together, these questions examine how aesthetic-functional congruence (rather than human-likeness or isolated features alone) impacts social evaluations of a robot design in wellbeing HRI contexts.

## 2 Materials and methods

### 2.1 Materials

This study employed visual stimulus materials consisting of (1) a short introductory video and (2) a set of four images depicting different visual design of the social robot Neffy (Figure 1). Neffy is a small tabletop breathing companion robot developed in the Communication Lab at the Berlin University of Applied Sciences, Germany, to support slow-paced breathing exercises for relaxation and stress reduction (Buchem et al., 2026a). The robot guides users through rhythmic breathing by combining gentle vertical motion, soft light signals, and a calm interaction style. To enable an embodied experience of breathing, Neffy moves its upper part of the torso upwards during inhalation and downwards during exhalation, enabling users to synchronize their breathing with the robot's motion. Rather than evaluating performance, Neffy is designed as a supportive companion that guides slow-paced breathing (Thomas et al., 2025; Buchem et al., 2026b,a).

**Figure 1:**
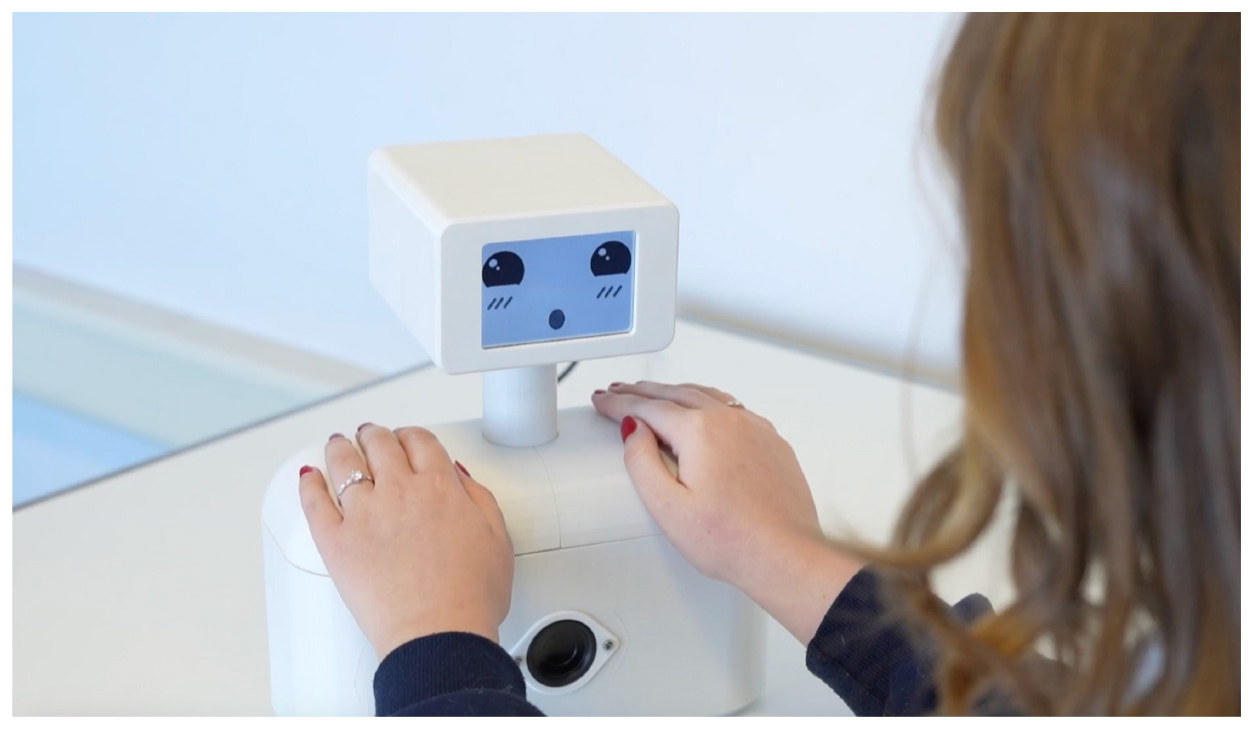
Screenshot of the introductory video with the original Neffy robot.

The visual design of the Neffy robot variants for the study was derived from classifications of robot embodiment types including anthropomorphic (human-like), zoomorphic (animal-like), mechanomorphic (machine-like), which can be further categorized into two broader categories of biomorphic (humanoid and zoomorphic) and mechanomorphic design (Fong et al., 2003; Mobed et al., 2024; Li et al., 2010). These categories reflect distinct embodiment strategies that provide different perceptual and functional cues and are known to influence users' expectations, perceptions, and evaluations of robot behavior and capabilities (Fong et al., 2003; Goetz et al., 2003; Syrdal et al., 2007). However, the design of the robot variants extends beyond morphological categories to incorporate distinct aesthetic styles, including cartoonish, toy-like, machine-like, and meditative aesthetics. The resulting four visual variants of the Neffy robot included: (A) cartoonish humanoid, (B) toy-like animal, (C) machine-like device, and (D) meditative humanoid, representing three biomorphic (two humanoid and one zoomorphic) and one mechanomorphic design category (see Figure 2). The playful humanoid version resembles a cute, cartoonish toy robot in blue and white color scheme with a cartoon-style human face on a screen with blushing cheeks and baby schema features including large eyes, small humanoid body with arms and feet, a warm yellow light at the front of the torso, all representing child-toy aesthetic. The zoomorphic version resembles a cute toy plush dog in warm beige and brown color scheme with animal-like features including floppy ears, snout, paws, tail, fur texture, blushing cheeks and a warm yellow light at the front of the torso, also representing animal-toy aesthetic. The mechanomorphic version resembles a device-like robot with a blue LED bar instead of a human-like or animal-like face, industrial gray housing with a black lens-like element at the front of the torso designed in a consumer electronics product aesthetic. The meditative humanoid version resembles a Buddha-like figure with a simplified human face with closed eyes and a gentle smile, soft-white rounded body, golden dome on top of the head and a warm yellow light at the front of the torso, all visually evoking the aesthetics of tranquility and mindfulness.

**Figure 2:**
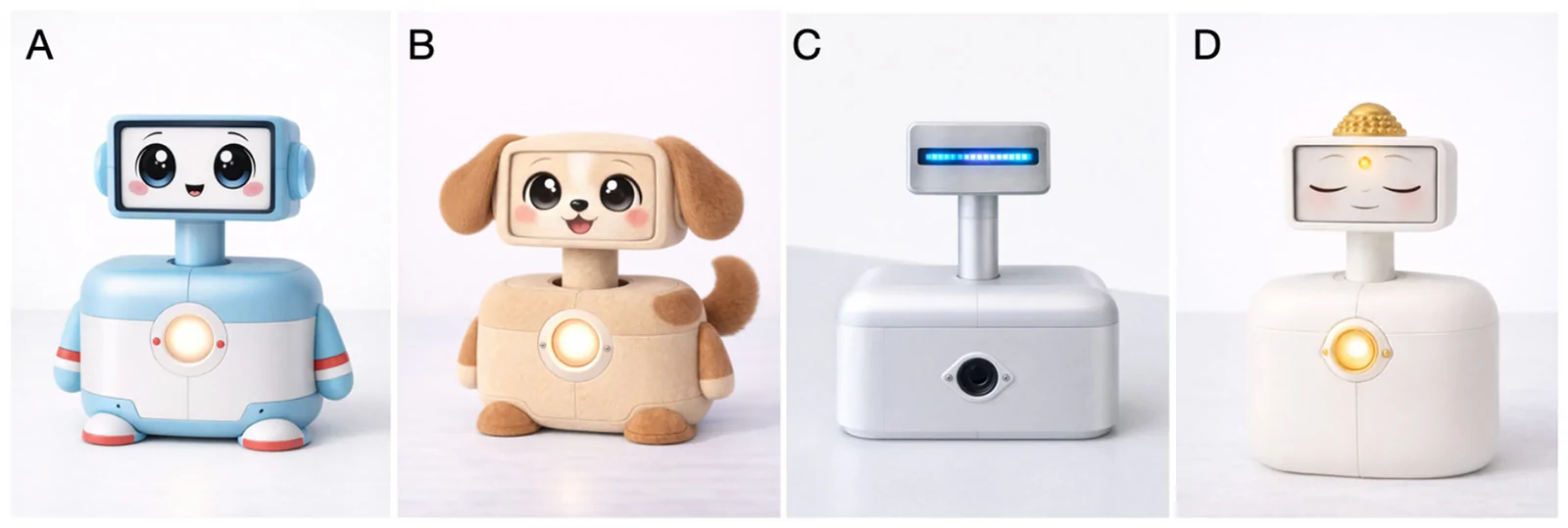
Aesthetic design styles: **(A)** Cartoonish humanoid design, **(B)** toy-like animal design, **(C)** machine-like device design, and **(D)** meditative humanoid design.

To isolate the effects of the visual design, the functional appearance of the robot was held constant across all four stimuli conditions. The Neffy robot in its original version was introduced through a short video demonstrating the breathing interaction. Participants did not directly interact with the robot but instead evaluated the four visual representations of different aesthetic designs of Neffy that varied in appearance while maintaining the same underlying functional design of the robot. This approach enabled a controlled investigation of how robot's aesthetic appearance influences perceived human-likeness, social evaluation across dimensions, and acceptance.

The visual stimuli were generated using a ChatGPT-based image generation pipeline in a multi-stage, iterative process. An original image of the Neffy robot served as a reference input to ensure consistency in proportions, structure, and identity across all generated variants. The design of the stimuli was informed by prior research on human-robot interaction and social perceptions of robots. In particular, design choices emphasized features associated with perceived warmth and trustworthiness, such as large, round eyes, upturned or neutral mouth shapes, and baby-schema features (Blow et al., 2006; Lehmann et al., 2013; Kalegina et al., 2018; Song and Luximon, 2024; Komatsu and Kamide, 2017; Onuki, 2014; Chen and Jia, 2023). The design strategy of the images aimed to avoid overly realistic human-like representations that could lead to mismatched expectations or negative reactions (Bradwell et al., 2021; Mori, 1970). The design aimed to balance biomorphic with abstract appearances over purely mechanical or highly realistic designs (De Graaf Allouch et al., 2015; Xiao et al., 2025; Schnitzer et al., 2024).

To systematically guide the image generation process, a structured prompt design was employed, consisting of modular components describing (1) the conceptual category (e.g., human-like, animal-like, machine-like), (2) degree of anthropomorphism, (3) facial features and gaze (e.g., eye shape, pupil size, presence of eyelids), (4) representation of breathing (e.g., visual or implied through design elements), (5) material properties (e.g., soft fur, smooth mechanical surfaces), and (6) color-scheme and visual style. Additional dimensions included the level of morphological abstraction (e.g., toy-like vs. realistic representations) and the use of visual signifiers indicating interaction affordances, e.g., sufficient flat surface for palm placement (Gibson, 1979; Norman, 2013; Sareh and Loudon, 2024). This structured approach ensured consistency across generated images while allowing controlled variation of key appearance-related design features.

In the first stage, a human-AI co-creation process was used to explore a wide range of embodiment concepts based on iterative prompt development. Multiple variants were generated representing different design strategies, including baby-like human representations, animal-like plush toy forms, functional machine-like devices, and figures representing design aesthetic of spiritual and meditative figures (such as an angel, a Buddha). In the second stage, 10 candidate images were independently evaluated by four evaluators (co-authors of this paper) using a set of five rating criteria: (1) clarity of the intended embodiment category, (2) consistency with the original Neffy morphology, (3) aesthetic coherence, (4) preservation of the intended interaction affordances, and (5) suitability for a breathing companion application, which were rated on a five-point rating scale (1 = low, 5 = high). The five categories were defined as follows: (1) Clarity of the intended embodiment category was defined as the extent to which the candidate can be unambiguously recognized as representing its intended category: cartoonish humanoid, animal-like, machine-like, or meditative humanoid; (2) Consistency with the original Neffy morphology was defined as the extent to which the candidate preserves the defining physical structure and identity of the original Neffy robot despite changes in visual style; (3) Aesthetic coherence was defined as the extent to which the visual elements form a unified and internally consistent design corresponding to the intended aesthetic concept; (4) Preservation of the intended interaction affordances was defined as the extent to which the design visually and physically communicates that a user can place both hands on the robot's upper body or shoulders and follow its breathing movement. Table 1 summarizes the descriptive statistics (*M*, SD) for the four best rated variants based on their overall scores. The evaluation forms completed by all four expert raters, together with the detailed ratings for all 10 image variants, are provided in Supplementary material S1.

**Table 1:** Descriptive statistics for the four highest-rated image variants (*N* = 4 expert raters).

Evaluation category	Image 1 Machine Like	Image 3 Animal Like	Image 5 Cartoonish Humanoid	Image 9 Meditative Humanoid
Overall	4.35 (0.72)	4.35 (1.05)	4.00 (1.51)	4.70 (0.35)
Clarity	5.00 (0.00)	5.00 (0.00)	4.25 (0.96)	4.75 (0.50)
Consistency	4.50 (1.00)	4.00 (2.00)	4.00 (2.00)	5.00 (0.00)
Coherence	5.00 (0.00)	5.00 (0.00)	4.00 (1.41)	4.75 (0.50)
Affordance	4.00 (1.41)	3.75 (1.89)	4.00 (1.41)	4.25 (0.50)
Suitability	3.25 (1.50)	4.00 (1.41)	3.75 (1.89)	4.75 (0.50)

Values are presented as Mean (Standard Deviation). Ratings were provided by four expert raters on a 5-point Likert scale, with higher scores indicating more positive evaluations. The four variants presented in the table achieved the highest overall ratings across the five evaluation criteria.

Images 1 and 3 were rated particularly highly for clarity (*M* = 5.00, SD = 0.00) and coherence (*M* = 5.00, SD = 0.00), while image 9 was rated particularly highly for consistency (*M* = 5.00, SD = 0.00) with full agreement among the raters in these criteria. Image 5 demonstrated a more balanced evaluation profile across all criteria with consistently good ratings for clarity (*M* = 4.25, SD = 0.96), consistency (*M* = 4.00, SD = 2.00), coherence (*M* = 4.00, SD = 1.41), affordances (*M* = 4.00, SD = 1.41).

The ratings served as an internal design selection procedure to identify representative stimuli for the subsequent experiment rather than as empirical data for hypothesis testing. Final image selection was based on a team discussion considering both quantitative and qualitative evaluations of the images. The selected variants were then further refined to ensure consistent proportions and visual comparability across conditions. To enhance comparability and maintain consistency with the interaction context, each robot variant was embedded into the same human-robot interaction context corresponding to the video used in the first part of the survey. For this purpose, a screenshot from the introductory video used in the survey was employed as a background context for prompt design, and each robot version was composited into the same scene. This resulted in a set of images depicting a human interacting with each design version of the Neffy robot in identical settings (see Figure 2).

This multi-stage generation process ensured controlled variation of robot appearance and interaction context. By holding the functional affordances constant while systematically varying visual design aesthetic, the stimulus design allowed for isolating appearance and function. The Supplementary Material S2 includes exact prompts for reproducibility.

### 2.2 Study design

This study employed a quantitative within-subject design to investigate how different visual designs of the robot influenced social perceptions of warmth and competence, perceived human-likeness, perceived enjoyment and behavioral intention as elements of the technology acceptance model as well as human-likeness, social presence and trustworthiness. Participants evaluated four versions of the Neffy robot presented in a randomized order. All participants viewed the same introductory video demonstrating a breathing interaction and subsequently evaluated each version individually and in pairwise comparisons. In this way, the analysis included both absolute ratings and comparative ratings of pairs of robot versions.

#### 2.2.1 Participants

Participants were recruited via the online platform Prolific. A total of *N* = 126 participants completed the study. Twenty participants were excluded due to incomplete responses, resulting in a final sample of *N* = 106 participants. Participants reported age, gender, country, and primary language, as well as experience with computers, breathing exercises, and meditation, and their general interest in robots. These variables were included to account for individual differences in familiarity and attitudes toward technology and wellbeing. To keep the survey concise, we did not employ multi-dimensional measures of attitudes toward robots, such as the MRAS (Ninomiya et al., 2015) or the GAToRS scale (Koverola et al., 2022). Participants ranged in age from 18 to 79 years (*M* = 38.97, SD = 12.58). The sample included 58 male and 45 female participants. The majority reported English as their primary language (*n* = 104), with most residing in the United States (*n* = 65) and the United Kingdom (*n* = 31), alongside a smaller number of participants from other countries. Participants reported moderate experience with computers (*M* = 3.99, SD = 1.05), lower experience with breathing exercises (*M* = 2.64, SD = 1.04) and meditation (*M* = 2.52, SD = 1.15), and moderate interest in robots (*M* = 3.21, SD = 1.07). These variables were collected to characterize the sample and to account for potential differences in familiarity with technology and wellbeing practices.

#### 2.2.2 Measures

*Perceived warmth and competence* were assessed using six-item Likert scales (1 = low, 5 = high) based on the Stereotype Content Model (Sevillano and Fiske, 2016). Warmth (W) items included: friendly, well-intentioned, trustworthy, warm, good-natured, and sincere. Competence (C) items included: competent, confident, capable, efficient, intelligent, and skillful. Internal consistency was high across robot versions: human-like (W α = 0.93, C α = 0.92), animal-like (W α = 0.91, C α = 0.93), machine-like (W α = 0.94, C α = 0.95), and symbolic (W α = 0.94, C α = 0.93).

*Technology acceptance* was measured using selected constructs from the Technology Acceptance Model (Davis, 1989; Venkatesh and Davis, 2000; Venkatesh et al., 2003), focusing on perceived enjoyment (ENJ) and behavioral intention to use (BI), which are appropriate for non-interactive evaluation contexts. Participants rated six items (1 = strongly disagree, 5 = strongly agree), including statements such as “Using this robot could be enjoyable” and “I would use this robot regularly.” Reliability was high across all conditions: human-like (ENJ α = 0.93, BI α = 0.94), animal-like (ENJ α = 0.93, BI α = 0.94), machine-like (ENJ α = 0.95, BI α = 0.92), and symbolic (ENJ α = 0.92, BI α = 0.95).

*Relative perceptions* were assessed using forced-choice pairwise comparisons across all versions (see Figure 3). Participants completed pairwise comparison tasks between two out of the four robot versions (see Figure 4). For each robot pair, participants selected the version that better matched a specific criterion. The pairwise measures included perceived social presence (“Which robot feels more present?”), perceived trustworthiness (“Which robot feels more trustworthy?”), perceived human-likeness (“Which robot is more human-like?”), breathing interaction preference (“Which robot would you prefer to practice breathing with?”), and overall preference (“Which robot do you like more?”). These measures were included to capture comparative perceptions related to social presence, trustworthiness, perceived human-likeness, behavioral intention, and general evaluation of the robot designs derived from research on social perceptions in human-robot interaction, including the CASA paradigm (Nass et al., 1994), anthropomorphism (Mergner et al., 2019), and user experience with hedonic and pragmatic qualities (Hassenzahl and Tractinsky, 2006; Hassenzahl, 2018).

**Figure 3:**
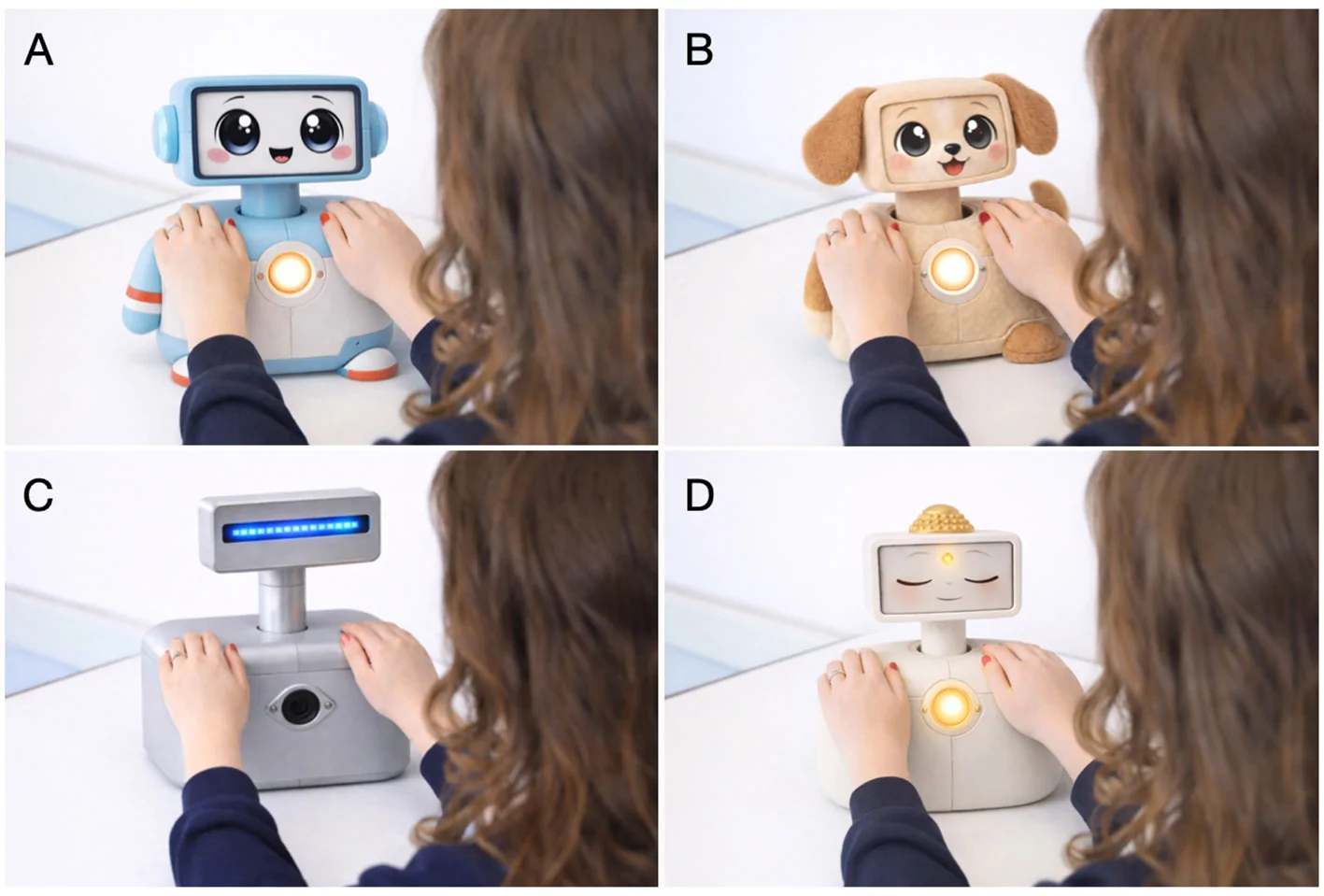
Images used in the study showing a human interacting with each version of the robot. Aesthetic design styles: **(A)** Cartoonish humanoid design, **(B)** Toy-like animal design, **(C)** Ma-chinelike device design, and **(D)** Meditative humanoid design.

**Figure 4:**
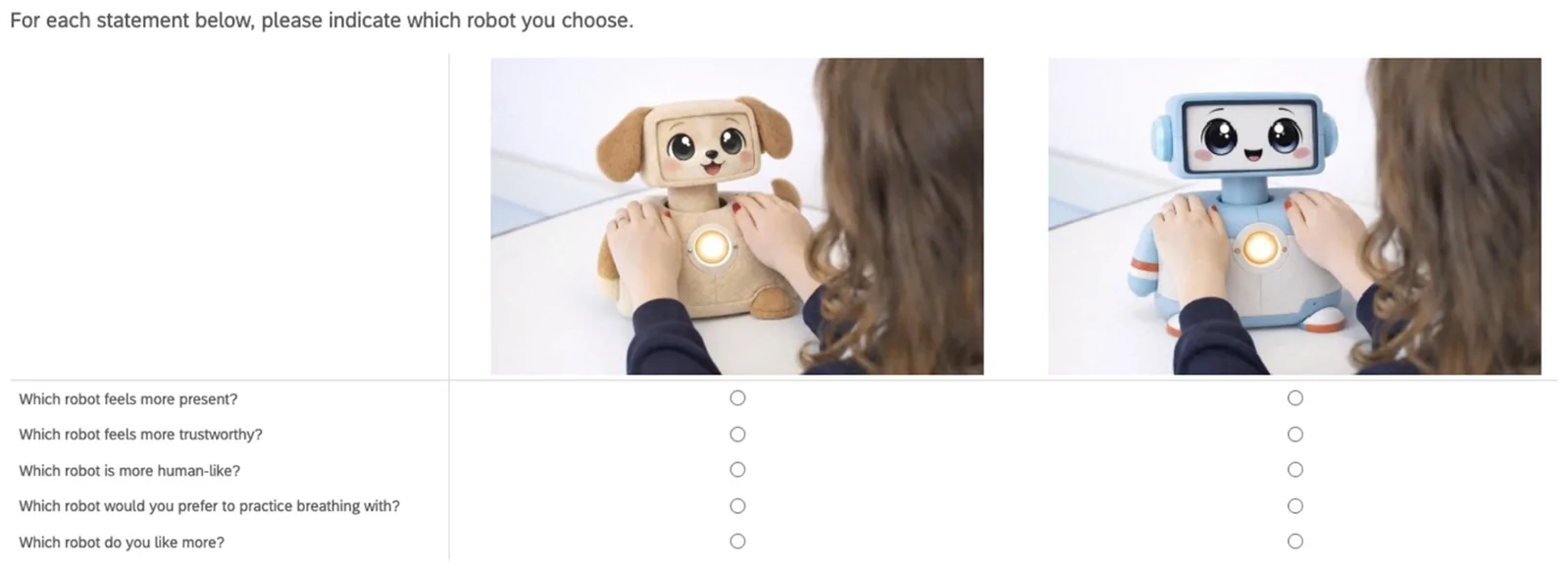
Pairwise comparison of two robot versions (here: animal-like vs. cartoonish humanoid).

#### 2.2.3 Procedure

The participants completed an online survey created in Qualtrics. After providing informed consent, participants were introduced to the Neffy robot and its function as a breathing companion for relaxation and stress reduction. Participants first watched a short video (9 s) introducing the Neffy robot and demonstrating a brief breathing interaction. The video explained that Neffy supports slow-paced breathing exercises for relaxation and stress reduction through gentle movement, light cues, and a calm voice. After viewing the clip, participants evaluated the four different visual designs of Neffy. The four robot versions were presented in random order for all participants. Participants evaluated each design independently using the same set of survey items before proceeding to the next design. Each design version was presented separately with an image and evaluated using multiple survey scales. Perceived warmth and perceived competence were measured using six items adapted from the Stereotype Content Model (SCM; Sevillano and Fiske, 2016). Technology acceptance including behavioral intention to use the robot were assessed using items adapted from the Technology Acceptance Model (TAM; Davis, 1989; Venkatesh and Davis, 2000; Venkatesh et al., 2003). After individual ratings, participants completed pairwise comparison tasks across all versions, which again were presented in random order. For each robot pair, participants selected which version felt more present, more trustworthy, more human-like, preferred for practicing breathing with, and more likable overall. Finally, demographic information and background variables were collected, including age, gender, country of residence, primary language, computer experience, experience with breathing exercises, experience with meditation or mindfulness, and general interest in robots.

#### 2.2.4 Data analysis

Data were analyzed in R. Raw data were exported from Qualtrics, and incomplete responses were removed if participants had not completed all rating blocks and pairwise comparison tasks. Likert-scale items were coded numerically (1–5), a unique participant identifier (PID) was assigned, and the data were structured in two formats: a long-format dataset for rating data (PID × Version) including all scale items, and a dataset for pairwise comparisons (PID × Pair × Question) to enable aggregation of comparison outcomes.

##### 2.2.4.1 Composite scores and reliability

Composite scores for warmth, competence, perceived enjoyment, and behavioral intention were computed as the mean of their respective items. Internal consistency was uniformly high across all scales and robot versions (Cronbach's α >0.90 in all 16 scale-by-version combinations), supporting the use of aggregated scores.

##### 2.2.4.2 Descriptive statistics and assumption checks

Descriptive statistics including means, standard deviations, medians, interquartile ranges, and 95% confidence intervals were calculated for each variable and robot version. Prior to inferential analyses, data quality and distributional assumptions were examined. Outliers were identified at both the overall and within-person levels. Normality of residuals was assessed using Shapiro–Wilk tests, which indicated significant violations for warmth, enjoyment, and behavioral intention across all versions (all *p* < 0.001); competence residuals were non-normal for two of four versions. Sphericity assessment with Mauchly's test showed violation for all four variables. Therefore, linear mixed-effects models (LMMs) were selected as the primary analysis in preference to repeated-measures ANOVA with Greenhouse–Geisser correction.

##### 2.2.4.3 Primary analyses (RQ1)

Differences between design styles. To examine differences between the four robot design styles in warmth, competence, perceived enjoyment, and behavioral intention, LMMs were specified with robot version as a fixed effect and participant as a random intercept to account for the repeated-measures structure of the data. Omnibus model effects were characterized using F statistics and partial eta squared (η^2^) as effect size estimates. Omnibus effects were followed by *post-hoc* pairwise comparisons using estimated marginal means with Holm correction; Cohen's d was reported for all significant pairwise contrasts.

##### 2.2.4.4 Pairwise comparison analyses (RQ2)

Forced-choice pairwise comparison responses were recoded as binary outcomes per comparison, and win counts ranging from 0 to 3 were calculated for each robot version across five criteria: perceived social presence, trustworthiness, human-likeness, breathing companion preference, and overall liking. Differences in win counts between robot versions were tested using Friedman rank-sum tests, followed by *post-hoc* Wilcoxon signed-rank tests with Holm correction. This non-parametric approach was appropriate given the ordinal and comparative nature of the forced-choice data.

##### 2.2.4.5 Correlational analyses

Pearson correlations were computed across all outcome variables, i.e., warmth, competence, perceived enjoyment, behavioral intention, human-likeness, trustworthiness, and social presence, to examine the structure of associations between social evaluation and technology acceptance constructs across robot versions.

##### 2.2.4.6 Human-likeness as a predictor (RQ3)

Perceived human-likeness was operationalized as win counts per robot version (range 0–3) derived from the pairwise comparison item “Which robot is more human-like?” Differences between versions in perceived human-likeness were first tested using Friedman and *post-hoc* Wilcoxon tests. To examine the relationship between perceived human-likeness and the primary outcome variables (warmth, competence, perceived enjoyment, behavioral intention, trustworthiness, and social presence) a series of LMMs was specified with human-likeness win counts as a continuous predictor, robot version as an additional fixed effect, and participant as a random intercept. This approach enabled the integration of relative (pairwise) and absolute (Likert-scale) measures within a single framework. Model fit was assessed with marginal and conditional *R*^2^.

##### 2.2.4.7 Individual differences as covariates

To examine whether embodiment effects on the primary outcomes remained after accounting for individual differences, additional LMMs were estimated including four user-level covariates: robot interest, meditation experience, breathing experience, and computer experience, all mean-centered. Model fit was compared between simple and full models using likelihood ratio tests, AIC, and BIC. These analyses addressed the potential confounding role of pre-existing attitudes and experiences with technology and wellbeing practices.

##### 2.2.4.8 Gender differences

Type III analyses of variance (ANOVA) with Satterthwaite approximation were conducted within the linear mixed-effects modeling framework to examine main effects of gender and robot embodiment as well as gender × embodiment interaction effects across the evaluated outcome variables.

## 3 Results

The distributions of participants' ratings across the four robot embodiments for warmth, competence, perceived enjoyment, and behavioral intention are illustrated in Figure 5. To examine the overall influence of robot design versions on social evaluations (warmth and competence) and acceptance outcomes (perceived enjoyment and behavioral intention), omnibus analyses were conducted across these four outcome dimensions.

**Figure 5:**
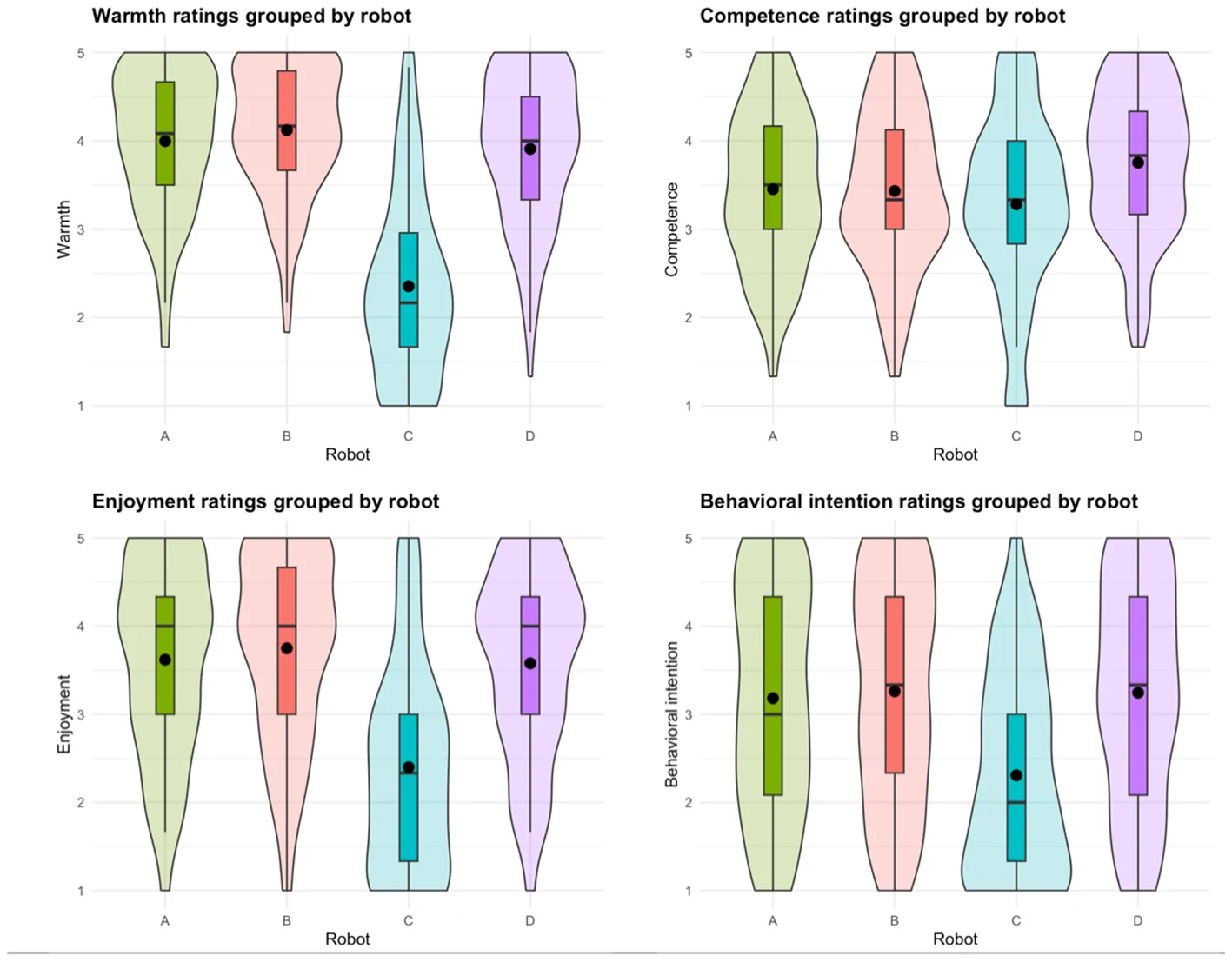
Violin plots showing participants' ratings of the four robot embodiments across the dimensions Warmth, Competence, Perceived Enjoyment, and Behavioral Intention. The black dots represent mean values, while the embedded boxplots indicate the median and interquartile range. Robot versions with the color legend: A (green): Cartoonish Humanoid; B (pink): Animal-Like; C (blue): Machine-Like; D (purple): Meditative Humanoid.

### 3.1 Warmth and competence (SMC)

*Warmth*. A linear mixed-effects model revealed a significant effect of robot design version on perceived warmth, *F*_(3, 315)_ = 154.69, *p* < 0.001, with a large effect size (η^2^ = 0.60), indicating that robot appearance strongly shaped participants' perceptions of friendliness, sincerity, and warmth. *Post-hoc* comparisons (Holm-corrected) showed that the cartoonish humanoid (A), animal-like (B), and meditative humanoid (D) versions were all rated significantly warmer than the machine-like version (C; all *p* < 0.0001), with large effect sizes for all three contrasts (A vs. C: *d* = 1.35; B vs. C: *d* = 1.46; D vs. C: *d* = 1.31). No significant differences in warmth were found among the A, B, and D versions, with all pairwise effect sizes negligible to small (A vs. B: *d* = 0.21; A vs. D: *d* = 0.12; B vs. D: *d* = 0.32), indicating that the two humanoid designs and the animal-like design were perceived as comparably warm. The animal-like version achieved the highest absolute warmth ratings (*M* = 4.12, SD = 0.73), followed closely by the cartoonish humanoid (*M* = 4.00, SD = 0.80) and the meditative humanoid (*M* = 3.91, SD = 0.85), while the machine-like version was rated substantially lower (*M* = 2.35, SD = 1.04). Notably, the two humanoid versions (A and D) did not differ significantly from each other in perceived warmth despite their markedly different aesthetic designs, version A cartoonish and playful, version D serene and meditative. This suggests that warmth perceptions in this context were driven primarily by aesthetic design cues (see Table 2).

**Table 2:** Descriptive statistics for warmth, competence, enjoyment, and behavioral intention across design versions: A cartoonish humanoid; B animal-like; C machine-like; D meditative humanoid. Values represent scores on a 5-point Likert scale, with higher values indicating stronger agreement.

Scale	A Cartoonish Humanoid *M* (SD)	B Animal Like *M* (SD)	C Machine Like *M* (SD)	D Meditative Humanoid *M* (SD)
Warmth	4.00 (0.80)^a^	4.12 (0.73)^a^	2.35 (1.04)^b^	3.91 (0.85)^a^
Competence	3.45 (0.87)^a^	3.43 (0.88)^a^	3.28 (1.02)^a^	3.75 (0.83)^b^
Enjoyment	3.62 (1.07)^a^	3.748 (1.06)^a^	2.40 (1.16)^b^	3.58 (1.03)^a^
Behavioral intention	3.18 (1.26)^a^	3.26 (1.25)^a^	2.31 (1.14)^b^	3.25 (1.25)^a^

Superscript letters indicate the results of Holm-corrected post-hoc pairwise comparisons within each scale. Means with the same superscript are not significantly different; means with different superscripts differ significantly at *p* < 0.01. Shapiro–Wilk *p* values indicate tests of normality.

*Competence*. A significant effect of robot design version on perceived competence was observed, *F*_(3, 315)_ = 9.23, *p* < 0.001, though with a substantially smaller effect size than warmth (η^2^ = 0.08), indicating that competence perceptions were less sensitive to robot appearance variation compared to warmth. *Post-hoc* comparisons revealed a clear distinction between the meditative humanoid version (D) and all other versions: D was rated significantly more competent than the cartoonish humanoid (*d* = 0.40; *p* = 0.0048), the animal-like version (*d* = 0.40; *p* = 0.0028), and the machine-like version (*d* = 0.47; *p* < 0.0001), with small effect sizes in all three cases. No significant pairwise differences were observed among the cartoonish humanoid, animal-like, and machine-like versions, with all pairwise effect sizes negligible (A vs. B: *d* = 0.03; A vs. C: *d* = 0.15; B vs. C: *d* = 0.13), indicating that these versions were perceived as comparably competent. The dissociation between the two humanoid versions is particularly noteworthy here. While the cartoonish humanoid (A) and meditative humanoid (D) were rated equivalently warm, they diverged significantly on competence, with the meditative humanoid rated higher (*M* = 3.75, SD = 0.83) than the cartoonish humanoid (*M* = 3.45, SD = 0.87). This pattern suggests that the serene, simplified aesthetic of the meditative humanoid communicated greater functional reliability and capability, possibly because of schema congruence and the activation of schemas associated with composed, serene meditation rather than the playful, child-like associations of the cartoonish design (see Table 2).

### 3.2 Perceived enjoyment and behavioral intention to use

#### 3.2.1 Perceived Enjoyment

Robot design version significantly influenced perceived enjoyment, *F*_(3, 315)_ = 76.56, *p* < 0.001, with a large effect size (η^2^ = 0.42). *Post-hoc* comparisons showed that the cartoonish humanoid (A), animal-like (B), and meditative humanoid (D) versions were all rated significantly more enjoyable than the machine-like version (C; all *p* < 0.0001), with large effect sizes for all three contrasts (A vs. C: *d* = 1.04; B vs. C: *d* = 1.08; D vs. C: *d* = 0.97). Differences among the A, B, and D versions were negligible and non-significant (all d < 0.20). No significant differences in enjoyment were observed among the A, B, and D versions. The animal-like version received the highest enjoyment ratings (*M* = 3.75, SD = 1.06), followed by the cartoonish humanoid (*M* = 3.62, SD = 1.07) and the meditative humanoid (*M* = 3.58, SD = 1.03), while the machine-like version was rated considerably lower (*M* = 2.40, SD = 1.16). As with warmth, both humanoid designs (A and D) did not differ significantly from each other in perceived enjoyment, despite their distinct aesthetic designs. The convergence in warmth and enjoyment for both human-like versions combined with the divergence in competence with higher ratings for the meditative humanoid designs suggests that human-like appearance and meditative aesthetics occupy distinct positions in the design space.

#### 3.2.2 Behavioral Intention

Robot design versions significantly influenced behavioral intention to use the robot, *F*_(3, 315)_ = 37.68, *p* < 0.001, with a medium effect size (η^2^ = 0.26). *Post-hoc* comparisons showed that the cartoonish humanoid (A), animal-like (B), and meditative humanoid (D) versions were all rated significantly higher in behavioral intention compared to the machine-like version (C; all *p* < 0.0001), with moderate to large effect sizes (*d* = 0.68, 0.77, and 0.84, respectively). No significant differences were found among the A, B, and D versions in relation to the behavioral intention.

### 3.3 Pairwise comparisons

Pairwise comparison analyses revealed significant differences across all evaluated criteria (see Table 3). A consistent pattern emerged across measures: the machine-like version (C) was significantly outperformed by all three biomorphic designs on every criterion, while meaningful distinctions among the cartoonish humanoid (A), animal-like (B), and meditative humanoid (D) were criterion-specific. The mean win counts from the pairwise comparisons across perceived human-likeness, social presence, trustworthiness, and breathing interaction preference are shown in Figure 6.

**Table 3:** Mean win counts for pairwise comparisons in social perception and preference measures.

Pairwise measure	A Cartoonish Humanoid ranking	B Animal Like ranking	C Machine Like ranking	D meditative Humanoid ranking
Human-likeness	2.27^a^ (0.59)	1.18^b^ (0.85)	0.34^c^ (0.72)	2.21^a^ (0.86)
Social presence	2.08^a^	1.72^b^	0.39^c^	1.82^ab^
Trustworthiness	1.80^a^	1.74^a^	0.46^b^	2.00^a^
Breathing interaction preference	1.79^a^	1.67^a^	0.46^b^	2.08^a^
Overall design preference	1.81^a^	1.81^a^	0.41^b^	1.97^a^

Values represent the average number of times each design was selected across its three pairwise comparisons for each measure. Scores could range from 0 to 3, with higher values indicating that the design was selected more frequently relative to the other robot versions. Superscript letters indicate Holm-corrected *post-hoc* Wilcoxon signed-rank comparisons following a significant Friedman test. Values sharing the same super script letter do not differ significantly; values with different superscript letters differ significantly at ab *p* < 0.001.

**Figure 6:**
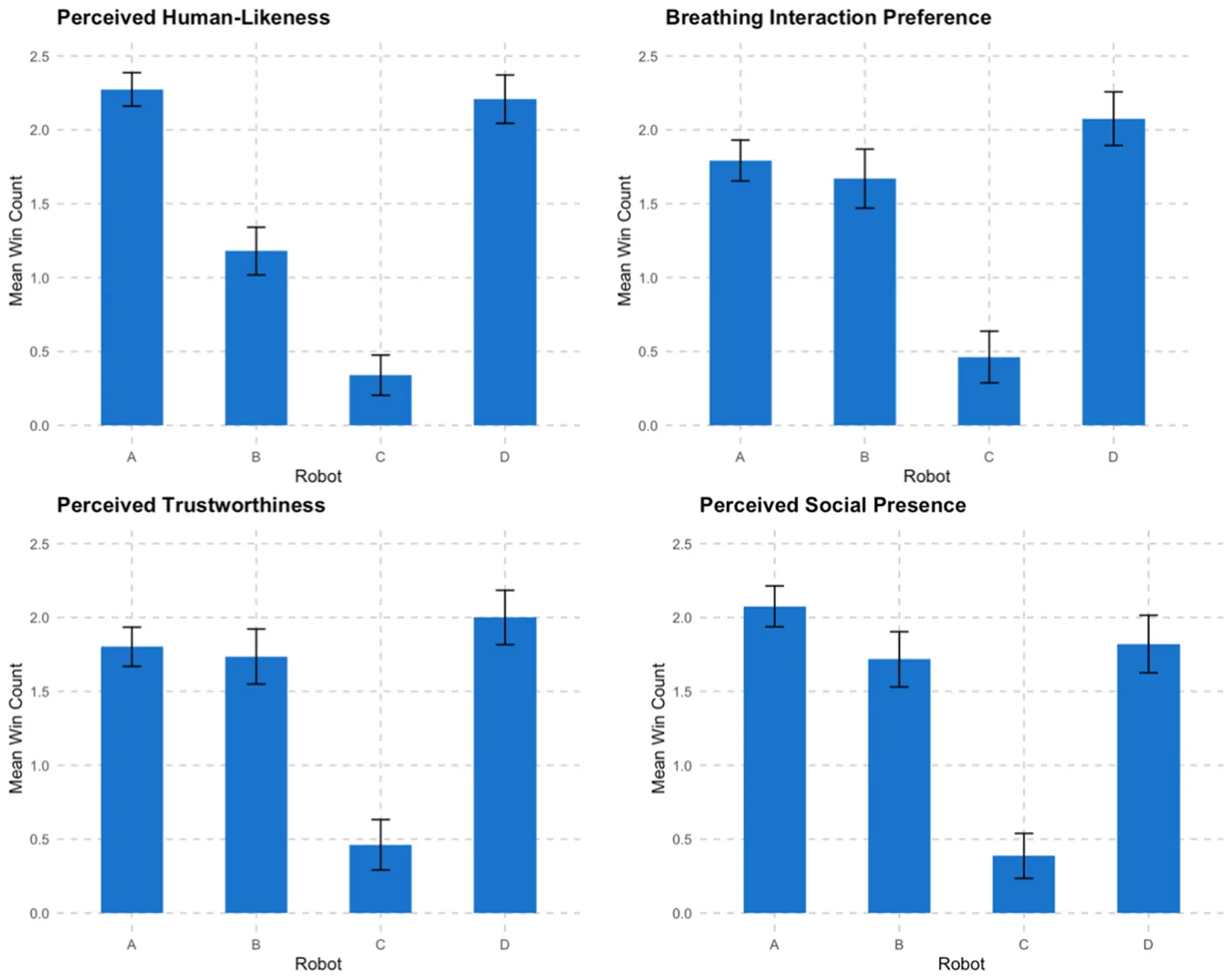
Mean win counts for pairwise comparison of perceived human-likeness, perceived social presence, perceived trustworthiness, breathing interaction preference across the design versions.

#### 3.3.1 Perceived human-likeness

Perceived human-likeness results showed this ranking: A (2.27) > D (2.21) > B (1.18) > C (0.34). Both humanoid versions were rated significantly more human-like than both the animal-like and machine-like versions (all *p* < 0.001), while no significant difference was found between the two humanoid designs (A vs. D, *p* = 0.565). This finding is theoretically important as despite the markedly different aesthetics of both humanoid versions participants perceived the two humanoid designs as equivalently human-like. This is in line with higher ratings in competence and trustworthiness of the meditative humanoid and points to the role of aesthetic-functional congruence in shaping these evaluations.

#### 3.3.2 Perceived social presence

For perceived social presence, a similar pattern emerged: The cartoonish humanoid version achieved the highest mean win counts (*M* = 2.08), followed by the meditative humanoid design (*M* = 1.82). Mean win counts for all four design versions showed the following ranking: A (2.08) > D (1.82) > B (1.72) > C (0.39). *Post-hoc* Wilcoxon signed-rank tests confirmed that both humanoid versions and the animal-like version were all rated significantly higher in social presence than the machine-like version (all *p* < 0.001). Notably, the cartoonish humanoid (A) was the only version to achieve a significant advantage over the animal-like version (*p* < 0.001), while the difference between the two humanoid designs (A vs. D) was not significant. This suggests that the cartoonish humanoid design might have communicated a stronger sense of social presence than the animal-like and meditative humanoid.

#### 3.3.3 Perceived trustworthiness

For trustworthiness, the meditative humanoid version (D) achieved the highest mean win counts (*M* = 2.00), followed by the cartoonish humanoid (*M* = 1.80). The ranking of the four design versions based on mean win counts was: D (2.00) > A (1.80) > B (1.74) > C (0.46). *Post-hoc* comparisons indicated that all three non-mechanomorphic versions were rated significantly more trustworthy than the mechanomorphic version (all *p* < 0.001), while no significant differences were observed among A, B, and D. However, the reversed ranking between A and D versions in social presence and trustworthiness, where the cartoonish humanoid (A) scored higher on social presence but the meditative humanoid (D) scored higher on trust, is noteworthy. Although neither humanoid design significantly outperformed the other on trustworthiness, the meditative humanoid achieved the highest absolute win count, consistent with its superior competence ratings in the Likert-scale analyses and suggesting that this aesthetic design may have communicated greater fit for task in the sense of aesthetic-functional congruence.

#### 3.3.4 Breathing interaction preference

For breathing interaction preference, the ranking was: D (2.08) > A (1.79) > B (1.67) > C (0.46). Both humanoid versions and the animal-like version were all preferred significantly more often than the machine-like version (all *p* < 0.001), with no significant differences among A, B, and D. The descriptive ranking is again informative as the meditative humanoid achieved the highest win count for breathing companion preference, corresponding to its higher rankings in trustworthiness and competence. The meditative humanoid was preferred, though not significantly, as a breathing companion over the cartoonish humanoid, which suggests that the aesthetics of version D was perceived as more contextually appropriate for a slow-paced breathing application, suggesting a higher aesthetic-functional congruence.

#### 3.3.5 Overall design preference

For overall design preference, the ranking was: D (1.97) > A (1.81) = B (1.81) > C (0.41). All three biomorphic versions were preferred significantly more often than the machine-like version (all *p* < 0.001), while no significant differences were observed among A, B, and D. The tie between the cartoonish humanoid and animal-like version in overall liking, combined with the meditative humanoid's numerical lead, suggests that general aesthetic appeal was distributed broadly across the three biomorphic designs, with no single design dominating hedonic preference.

Overall, two overarching patterns emerged. First, the machine-like version was consistently and significantly outperformed by all biomorphic designs, supporting and extending the Likert-scale findings presented in Tables 1, 2. Second, within the three biomorphic designs (A, B, D), the two humanoid versions (A and D) showed a criterion-specific differentiation, with the cartoonish humanoid (A) scoring higher on social presence, and the meditative humanoid scoring higher on trustworthiness, breathing companion preference, and overall design preference. However, none of the differences between biomorphic versions reached statistical significance. This pattern of numerical but non-significant advantages for the meditative humanoid across multiple functionally relevant criteria is consistent with the hypothesis that aesthetic-functional congruence, rather than human-likeness per se, drives user preferences in wellbeing-oriented HRI contexts.

### 3.4 Correlation Analysis Results

To examine the structure of associations among outcome variables, Pearson correlations were computed across warmth, competence, trustworthiness, social presence, perceived enjoyment, and behavioral intention. Results are presented in Table 4. The strongest associations emerged between warmth, trustworthiness, and social presence on one hand, and acceptance outcomes on the other hand. Warmth correlated strongly with enjoyment (*r* = 0.79, *p* < 0.001) and behavioral intention (*r* = 0.66, *p* < 0.001), indicating that design versions perceived as warmer were also evaluated as more enjoyable and elicited stronger willingness to use the robot for slow-paced breathing. Trustworthiness showed similarly strong relationships with behavioral intention (*r* = 0.76, *p* < 0.001) and enjoyment (*r* = 0.73, *p* < 0.001), suggesting that trust may play a central role in users' willingness to engage with a robot supporting slow-paced breathing. Social presence was also positively associated with enjoyment (*r* = 0.68, *p* < 0.001) and behavioral intention (*r* = 0.70, *p* < 0.001), indicating that robot designs experienced as more socially present were perceived as more engaging and acceptable interaction partners. Warmth and trustworthiness were themselves strongly intercorrelated (*r* = 0.71, *p* < 0.001), supporting the close relationship between emotional and relational evaluations in HRI contexts. Competence showed a weaker correlation pattern, with lower associations with enjoyment (*r* = 0.44, *p* < 0.001), behavioral intention (*r* = 0.47, *p* < 0.001), and social presence (*r* = 0.39, *p* < 0.001), and the weakest correlation with human-likeness of all outcomes (*r* = 0.18). These findings suggest that competence evaluations may be more independent from emotional and relational perceptions, which positions competence as the most functionally independent evaluation outcomes, consistent with the small effect of design version on competence (η^2^ = 0.08) reported in the sections below.

**Table 4:** Correlations between social evaluation and acceptance variables.

Variable	1	2	3	4	5	6	7
1. Warmth	—						
2. Competence	0.53^***^	—					
3. Perceived enjoyment	0.79^***^	0.64^***^	—				
4. Behavioral intention	0.66^***^	0.65^***^	0.87^***^	—			
5. Human-likeness	0.46^***^	0.18^*^	0.38^***^	0.29^***^	—		
6. Trustworthiness	0.52^***^	0.24^**^	0.47^***^	0.38^***^	0.65^***^	—	
7. Social presence	0.50^***^	0.23^**^	0.45^***^	0.35^***^	0.67^***^	0.77^***^	—

^*^p < 0.05, ^**^p < 0.01, ^***^p < 0.001.

### 3.5 Human-likeness and social evaluation of robot design

To investigate how perceived human-likeness related to social evaluation and technology acceptance across the four design versions, a series of linear mixed-effects models (LMMs) were conducted with participant as a random intercept, robot design version as a fixed effect, and human-likeness win counts as a continuous predictor. This approach enabled the integration of relative (pairwise) and absolute (Likert-scale) measures within a unified analytical framework.

#### 3.5.1 Human-likeness and warmth

Perceived human-likeness was a significant positive predictor of warmth (β = 0.204, *p* < 0.001, 95% CI [0.121, 0.287]), indicating that design versions perceived as more human-like were also rated as warmer with warmth ratings increased by 0.204 points for each additional human-likeness win count (Table 5). The model explained a substantial proportion of variance in warmth ratings (marginal *R*^2^ = 0.431; conditional *R*^2^ = 0.649). This suggests that perceived human-likeness and design version jointly accounted for much of the individual variation in warmth evaluations. The correlation between human-likeness and warmth was strong (*r* = 0.66). However, this relationship requires careful interpretation in light of the design version results: despite the two humanoid versions (A and D) being rated as human-like, the animal-like version (B) achieved the highest absolute warmth ratings (*M* = 4.12, SD = 0.73), followed by the cartoonish humanoid (*M* = 4.00, SD = 0.80) and meditative humanoid (*M* = 3.91, SD = 0.85). The linear mixed-effects model demonstrated a strong overall effect of robot design on warmth, *F*_(3, 315)_ = 154.69, *p* < 0.001, with a large effect size (η^2^ = 0.60). *Post-hoc* comparisons revealed that all three non-mechanomorphic versions were rated significantly warmer than the mechanomorphic version (all *p* < 0.001), while no significant differences emerged among both humanoid and the animal-like versions. A contrast analysis confirmed that the animal-like version was rated significantly warmer than the average of the remaining three versions, *t*_(315)_ = 9.08, *p* < 0.001, with an estimated difference of 0.70 (SE = 0.077). This indicates that animal-like design evoked warmth perceptions at least as strong as, and on the descriptive level exceeding, those produced by either humanoid design. This suggests that warmth was driven by shared biomorphic qualities rather than by human-likeness specifically.

**Table 5:** Linear mixed-effects models predicting warmth and competence from human-likeness.

Outcome Variable	β (Human-Likeness)	95% CI	*p*	Marginal *R*^2^	Conditional *R*^2^
Warmth	0.204	[0.121, 0.287]	< 0.001	0.431	0.649
Competence	0.136	[0.053, 0.218]	0.001	0.047	0.488

Positive β coefficients indicate that higher perceived human-likeness was associated with higher ratings on the respective outcome variable. Models included participant as a random intercept.

The large difference between marginal and conditional R^2^ for competence (ΔR^2^ = 0.441) indicates that individual differences between participants accounted for substantially more variance in competence evaluations than perceived human-likeness. This suggests that competence perceptions were impacted by participant-level factors.

#### 3.5.2 Human-likeness and competence

Perceived human-likeness also significantly predicted competence ratings (β = 0.136, *p* = 0.001, 95% CI [0.053, 0.218]), though the relationship was weaker than for warmth, both in regression coefficient and correlation (*r* = 0.46 vs. *r* = 0.66). Competence ratings increased by 0.136 points for each additional human-likeness win count (vs. 0.204 points increase for warmth). The model explained considerably less variance (marginal *R*^2^ = 0.047; conditional *R*^2^ = 0.488) compared to warmth. The meditative humanoid (D) received the highest competence ratings (*M* = 3.75, SD = 0.83), rated significantly more competent than the cartoonish humanoid (A: *M* = 3.45, SD = 0.87; *p* = 0.0048), animal-like (B: *M* = 3.43, SD = 0.88; *p* = 0.0028), and machine-like versions (C: *M* = 3.28, SD = 1.02; *p* < 0.001). The overall effect of design version on competence was small (η^2^ = 0.08). This suggests that competence perceptions were less impacted by appearance variation compared to warmth. The higher competence ratings of meditative compared to the cartoonish humanoid design, with both versions rated as equivalently human-like, cannot be explained by differences in perceived human-likeness, and instead points to the role of aesthetic design in communicating a functional fit for task, supporting the aesthetic-functional congruence hypothesis.

Figure 7 illustrates the relationships between perceived human-likeness win counts and warmth ratings (left) and competence ratings (right) across robot embodiments. Higher perceived human-likeness was associated with increased warmth (β = 0.204, *p* < 0.001) and competence ratings (β = 0.136, *p* = 0.001). In line with the preceding analyses, the meditative humanoid version (D) achieved similar or higher evaluations in trustworthiness, competence, and preference compared to a breathing companion compared to the cartoonish humanoid (A), despite similar perceived human-likeness. Colored points represent individual participant ratings for the four robot design versions (A–D).

**Figure 7:**
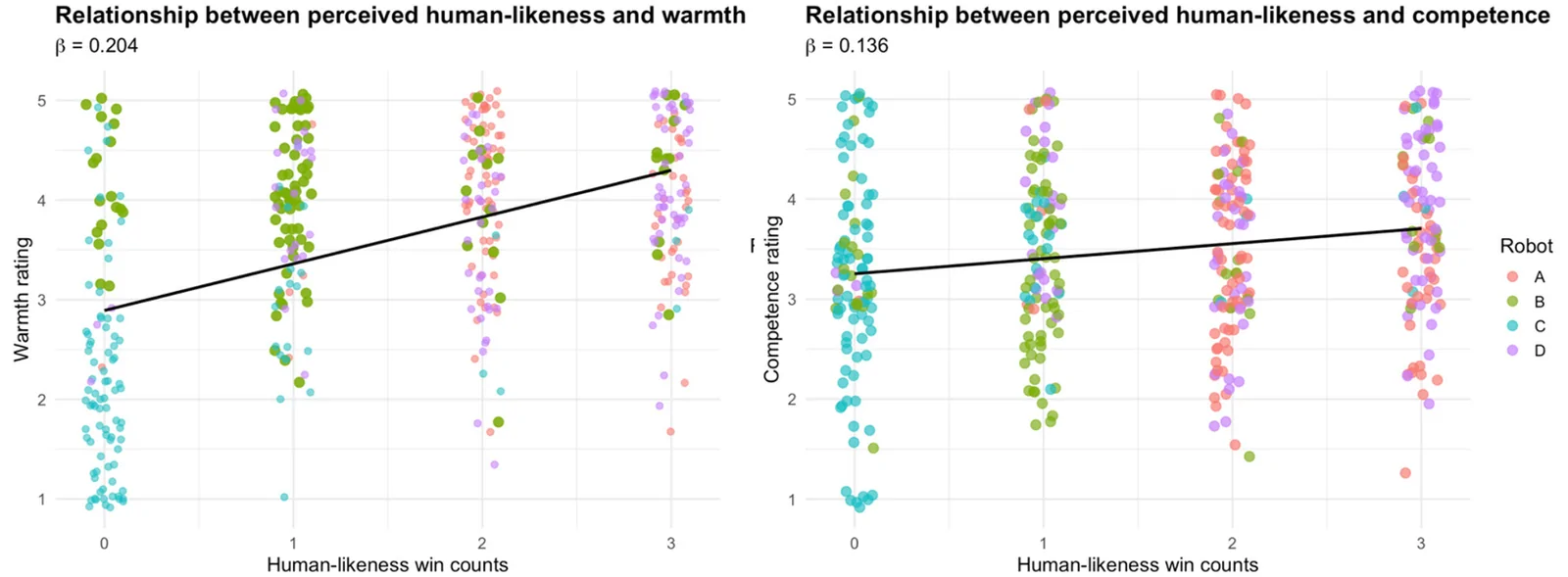
Relationships between human-likeness, warmth and competence. Robot versions with the color legend: A (green): Cartoonish Humanoid; B (pink): Animal-Like; C (blue): Machine-Like; D (purple): Meditative Humanoid.

#### 3.5.3 Human-likeness and all social evaluation outcomes

The results from the linear mixed-effects model (LMM) analysis across all primary outcome dimensions showed that perceived human-likeness was a significant positive predictor of all evaluated outcomes, with the strongest predictive relationships observed for social presence (β = 0.587, *p* < 0.001, marginal *R*^2^ = 0.514) and trustworthiness (β = 0.583, *p* < 0.001, marginal *R*^2^ = 0.485), followed by perceived enjoyment (β = 0.294, *p* < 0.001, marginal *R*^2^ = 0.237), behavioral intention (β = 0.257, *p* < 0.001, marginal *R*^2^ = 0.119), warmth (β = 0.204, *p* < 0.001, marginal *R*^2^ = 0.431), and competence (β = 0.136, *p* = 0.001, marginal *R*^2^ = 0.047). Perceived human-likeness most strongly predicted social presence (β = 0.587, *p* < 0.001) and trustworthiness (β = 0.583, *p* < 0.001). From the perspective of the CASA theory (Nass et al., 1994), this suggests that specific perceptual cues related to robot appearance triggered the activation of social-cognitive processes, which led to the attribution of human-like characteristics and enhanced perceptions of presence and trust, i.e., robot versions perceived as more human-like were also experienced as more socially present and trustworthy interaction partners. Table 6 summarizes the linear mixed-effects model results across the social evaluation and acceptance outcomes.

**Table 6:** Predicting social evaluation and acceptance outcomes from perceived human-likeness.

Outcome	β Human-Likeness	SE	95% CI	*p*	Marginal *R*^2^
Warmth	0.204	42	[0.121, 0.287]	< 0.001	0.431
Competence	0.136	42	[0.053, 0.218]	0.001	0.047
Trustworthiness	0.583	47	[0.486, 0.675]	< 0.001	0.485
Social presence	0.587	45	[0.491, 0.684]	< 0.001	0.514
Perceived enjoyment	0.294	51	[0.207, 0.381]	< 0.001	0.237
Behavioral intention	0.257	58	[0.164, 0.35]	< 0.001	0.119

Human-likeness scores were derived from pairwise comparison win counts. Positive β coefficients indicate that higher perceived human-likeness was associated with higher ratings on the respective outcome variable. Marginal *R*^2^ values represent variance explained by fixed effects only.

#### 3.5.4 The limits of human-likeness as an explanatory variable

In further analysis, individual differences were included as covariates. These differences encompassed robot interest, meditation experience, breathing experience, and computer experience. Among the four covariates included in the analyses, only participants' prior interest in robots emerged as a significant predictor. Computer experience, meditation experience, and breathing experience were not significantly associated with the evaluated outcome measures. The predictive effect of perceived human-likeness was no longer statistically significant for any of the four primary Likert-scale outcomes (warmth: β = 0.088, *p* = 0.210; competence: β = 0.068, *p* = 0.321; enjoyment: β = 0.057, *p* = 0.415; behavioral intention: β = 0.110, *p* = 0.152). In contrast, robot interest remained a significant positive predictor across all four outcomes (warmth: β = 0.224, *p* = 0.032; competence: β = 0.288, *p* = 0.016; enjoyment: β = 0.408, *p* < 0.001; behavioral intention: β = 0.556, *p* < 0.001). Model fit was significantly improved by the inclusion of covariates for competence [$\chi_{\left(4\right)}^{2}$ = 9.92, *p* = 0.042], enjoyment (*p* = 0.003), and behavioral intention (*p* = 0.0005). In contrast, no meaningful improvement in model fit was observed for warmth (*p* = 0.150), suggesting that warmth perceptions remained more strongly linked to design-related factors.

These findings suggest that pre-existing interest in robots played a more substantial role in shaping evaluations than perceived human-likeness once individual differences were accounted for. Moreover, these findings suggest that individual differences, such as interest in robots, may play a more substantial role in shaping social evaluations and acceptance of social robots supporting slow-paced breathing, rather than design characteristics alone. These findings are also consistent with the distinction between anthropomorphism and the CASA paradigm (Heyselaar, 2023). Our findings support the view that positive evaluations cannot be explained solely by perceived human-likeness. Table 7 summarizes the linear mixed-effects models examining the influence of perceived human-likeness and robot interest on warmth, competence, enjoyment, and behavioral intention after inclusion of user-related covariates.

**Table 7:** Effects of robot interest on social evaluation and acceptance variables.

Variables	Human-likeness after covariates	Robot interest	Model fit improvement
Warmth	β = 0.088, *p* = 0.210	β = 0.224, *p* = 0.032	ns
Competence	β = 0.068, *p* = 0.321	β = 0.288, *p* = 0.016	χ^2^ = 9.92, *p* = 0.042
Enjoyment	β = 0.057, *p* = 0.415	β = 0.408, *p* < 0.001	*p* = 0.003
Behavioral Intention	β = 0.110, *p* = 0.152	β = 0.556, *p* < 0.001	*p* = 0.0005

Linear mixed-effects models included participant as a random intercept. Robot interest was included as a covariate to examine the influence of individual differences.

### 3.6 Gender differences

To examine whether gender influenced social evaluation and acceptance outcomes, and whether gender moderated the effects of robot design version, additional linear mixed-effects models were conducted for each outcome variable with gender and robot design version as fixed effects and their interaction term included. For the majority of the evaluation outcomes, there were no significant effects of gender, with the exception of competence and behavioral intention. First, no significant main effect of gender on warmth was observed [*F*_(1, 101)_ = 1.64, *p* = 0.203], indicating that male and female participants did not differ meaningfully in their overall warmth evaluations of the robot designs. However, a small but significant main effect of gender emerged for competence ratings [*F*_(1, 101)_ = 4.42, *p* = 0.038], with female participants rating the robot designs as slightly more competent compared to male participants. The gender × design version interaction was non-significant [*F*_(3, 303)_ = 1.08, *p* = 0.359], indicating that the pattern of competence differences across design versions was consistent regardless of gender. The statistically significant main effect of gender should be interpreted with caution given the small overall effect of design version on competence (η^2^ = 0.08). Second, while no significant main effect of gender was found for enjoyment [*F*_(1, 101)_ = 1.17, *p* = 0.283] and behavioral intention [*F*_(1, 101)_ = 0.55, *p* = 0.459], the robot design version had a significant effect on behavioral intention [*F*_(3, 303)_ = 37.94, *p* < 0.001]. This finding suggests that some designs were associated with substantially greater willingness to use. Also, a significant interaction between gender and robot design version emerged [*F*_(3, 303)_ = 3.96, *p* = 0.009], indicating that the effect of design version on behavioral intention was not identical for male and female participants.

### 3.7 Qualitative analysis results

Participants were invited to provide optional open-ended comments at the end of the survey. 47% of participants (50 out of 106) provided written responses, ranging from brief evaluative remarks to more elaborated reflections on design preferences and personal relevance. Responses were analyzed using a combination of deductive and inductive thematic coding (Braun and Clarke, 2006). Deductive codes were derived from the theoretical models of the study. Inductive codes emerged from the data and captured themes not anticipated by the theoretical framework.

*Design and appearance preferences*. Participants frequently commented on the aesthetic qualities of the four design versions, with responses largely consistent with the quantitative findings. The machine-like version was the most frequently criticized, with participants describing it as “*cold*” and “*clinical*,” echoing the low warmth and enjoyment ratings of the machine-like version and supporting the interpretation that mechanomorphic design communicated affordances associated with technical functionality rather than emotional support (Norman, 2013). One participant described the machine-like version humorously as “*really funny, reminded me of a Dalek. EXTERMINATE*,” which captures the emotional distance evoked by strongly mechanomorphic aesthetics. The cartoonish humanoid was described by one participant as “*too childish*” and by another one as “*not very realistic*” suggesting that its playful aesthetic design may have activated a toy-related schema rather than a therapeutic or wellbeing schema, which aligns with the quantitative result that the cartoonish humanoid was rated lower on competence than the meditative humanoid despite similar perceived human-likeness. The animal-like version was described by one participant as “*too playful*.” The meditative humanoid version was implicitly or explicitly preferred by several participants as the most appropriate design, and was described along the lines of “*the best one*” in the context of a direct comparison across all four versions. One participant expressed a preference for the original robot shown in the demonstration video, noting: “*I actually preferred the robot in the initial short demonstration video*,” suggesting that the original design and possibly the interaction context may shape design preferences beyond appearance alone. Another participant suggested a preference for a different form factor, writing: “*I'd prefer a smaller robot overall*.” Such comments indicate that robot size, familiarity, emotional expressiveness, and perceived friendliness may influence user acceptance in wellbeing contexts.

*Perceived aesthetic-functional fit*. Several participants explicitly connected the robot concepts to personal health experiences or expressed enthusiasm about the potential of robot-assisted wellbeing technologies, reflecting activation of a wellbeing schema consistent with Schema Congruity Theory (Meyers-Levy and Tybout, 1989). One participant wrote: “*Wonderful idea. Exciting to see what assistance type of tools using AI/Robots would be coming out in the future*.” Others connected the concept directly to personal health contexts: “*I have long covid and asthma so this is something I'd find quite interesting*” and “*Very interesting concept especially since what I'm going through these robots would help my mindfulness*.” A further participant noted: “*I could see these support robots helping people with anxiety*.” These responses suggest that participants possibly activated a companion or therapeutic schema when evaluating the robots, perceiving them not merely as functional devices but as potential wellbeing support partners, consistent with the Partner Choice Framework (Campennì and Schino, 2014) and hedonic aspects of user experience (Hassenzahl, 2018). Several comments reflected spontaneous schema-based evaluations consistent with the theoretical framework of aesthetic-functional congruence proposed in this paper. Participants appeared to evaluate the robots not only on aesthetic grounds but in relation to their perceived fit with the functional support for slow-paced breathing for stress reduction. The preference for the meditative humanoid design among participants suggests that the applied aesthetic of a serene and composed meditative figure activated schemas associated with mindfulness, calm, and therapeutic support more readily than the cartoonish humanoid or mechanomorphic designs. This pattern is consistent with Goetz et al.'s (2003) finding which showed that users prefer robots whose appearance matches the sociability and seriousness of the task, and with Schema Congruity Theory, which posits that a moderate, resolvable incongruity, e.g., a robot that is neither a pure machine nor a human replica, but a recognizably calm and purposeful wellbeing companion, produces most favorable evaluations (Campbell and Goodstein, 2001).

The qualitative findings converge with and extend the quantitative results in three ways. First, they confirm that the machine-like design was perceived as emotionally cold and functionally mismatched with a wellbeing context, supporting its consistently low quantitative evaluations. Second, the findings suggest that the playful aesthetic of the cartoonish humanoid version may have activated toy-related rather than therapeutic schemas, providing a qualitative explanation for lower competence ratings of the cartoonish humanoid compared to the meditative humanoid. Third, the results of the qualitative analysis indicate that participants spontaneously evaluated the robots in terms of their perceived fit with the task for the support of slow-paced breathing for stress reduction, providing qualitative support for the aesthetic-functional congruence related to user evaluations of the robot.

## 4 Discussion

### 4.1 Human-likeness and social evaluation

The findings of this study confirm that perceived human-likeness played a significant role in shaping social evaluations of the Neffy robot designs. Across all primary evaluation outcomes, i.e., warmth, competence, trustworthiness, social presence, enjoyment, and behavioral intention, higher perceived human-likeness was associated with more positive evaluations, with the strongest predictive relationships observed for social presence (β = 0.587) and trustworthiness (β = 0.583). These patterns are consistent with the CASA paradigm (Nass et al., 1994), which proposes that humans automatically, and unconsciously, apply social rules and interpersonal expectations to technological systems displaying socially meaningful cues (Heyselaar, 2023). The strong associations between perceived human-likeness, social presence, and trustworthiness suggest that human-like design cues activated social-cognitive processes typically associated with human interaction, making the robots appear as meaningful and reliable social partners rather than mere functional devices.

The relation of warmth and competence to human-likeness is theoretically meaningful and consistent with the Stereotype Content Model (Sevillano and Fiske, 2016). Warmth showed the largest effect of design version (η^2^ = 0.60) and the strongest correlation with human-likeness (*r* = 0.66), reflecting the well-established finding that warmth judgments are formed rapidly on the basis of perceptual and interpersonal cues (Cuddy et al., 2007; Bergmann et al., 2012). Competence, by contrast, showed the smallest effect of design version (η^2^ = 0.08) and the weakest correlation with human-likeness (*r* = 0.46), consistent with SCM research showing that competence evaluations are more resistant to surface-level appearance variations and more dependent on contextual expectations (Bergmann et al., 2012). The large gap between marginal and conditional *R*^2^ for the competence model (Δ*R*^2^ = 0.441) suggests that individual differences between participants dominated competence evaluations, with robot appearance accounting for only a small proportion of variance. This dissociation between warmth and competence has some relevant implications for design, such as optimizing appearance for warmth and competence may require different strategies. Present findings suggest these strategies can diverge meaningfully even within the same morphological category.

The robot interest covariate finding deserves particular attention. When robot interest was included as a covariate, the effects of perceived human-likeness on all primary outcomes became non-significant, while robot interest remained significant across all four outcomes (all *p* < 0.05). This finding suggests that participants who were more interested in robots evaluated all designs more positively across the board, independent of the specific design version, which was a general positivity effect that masked the independent contribution of perceived human-likeness when not controlled.

The most important finding with respect to human-likeness is not its effect but its limits. When individual differences, specifically robot interest, were included as covariates, the predictive effect of perceived human-likeness on all four primary Likert-scale outcomes became non-significant, while robot interest remained a significant positive predictor across all outcomes (all *p* < 0.05). This indicates that a substantial portion of the variance previously attributed to perceived human-likeness reflected pre-existing attitudes toward robots rather than the design-specific effect of anthropomorphic appearance. This finding suggests that user-level factors may be at least as important as design-level factors in shaping evaluations.

### 4.2 Aesthetic-function congruence

The central and most novel finding of this study is that the two humanoid designs, i.e., the cartoonish humanoid (A) and the meditative humanoid (D), were perceived as equivalently human-like yet produced systematically different evaluations on competence, trustworthiness, and breathing companion preference, with the meditative humanoid consistently outperforming the cartoonish humanoid on these functionally relevant dimensions. This dissociation empirically supports the core argument of this paper, i.e., that aesthetic-functional congruence, i.e., the alignment or fit between a robot's aesthetic and functional design may be a more powerful explanation of user preferences than human-likeness or morphology alone. This interpretation is grounded in Schema Congruity Theory (Meyers-Levy and Tybout, 1989; Campbell and Goodstein, 2001; Gao et al., 2022), which proposes that individuals evaluate objects in relation to pre-existing cognitive schemas, with moderate and resolvable incongruity producing the most favorable evaluations.

The cartoonish humanoid, with its playful baby-schema features, possibly activated a toy schema, which may be engaging and likable while possibly activating care-giver and/or toy-play schemas, but seems to be incongruent with the composed, calm and serene aesthetics associated with a therapeutic slow-paced breathing practice for anxiety and stress reduction. One participant's qualitative comment that version A was “too childish” directly supports this interpretation. The meditative humanoid, by contrast, with its simplified face, closed eyes, gentle smile, and Buddhist-inspired golden dome, appears to have activated schemas associated with mindfulness, contemplation, and serene purposefulness. These qualities are congruent with the intended function of a breathing companion for anxiety and stress reduction. This finding aligns with the “appropriate anthropomorphism” (Goetz et al., 2003), i.e., sufficient social accessibility to feel relatable and trustworthy, without mismatched expectations toward explicitly human-like or toy-like designs.

The Form-Affordance-Function (FAF) framework (Sareh and Loudon, 2024) provides a complementary explanation. In this framework, visual form not only reflects functional properties but shapes perceived affordances, i.e., the interaction possibilities users read from appearance alone. The composed, simplified aesthetic of the meditative humanoid possibly communicated affordances of calm, focused, and purposeful engagement, aligned with the breathing synchronization function of the Neffy robot. The cartoonish humanoid possibly communicated affordances of playful, social, and child-directed interaction, which may be engaging but seemed less convincingly competent or trustworthy as a breathing-support tool. This is consistent with Norman's (2013) distinction between affordances and signifiers. The visual design of each robot functioned as a signifier, communicating not only how to interact with the robot but what kind of interaction and what kind of relationship was appropriate. The meditative humanoid's signifiers were congruent with a wellbeing context in ways the cartoonish humanoid were not, despite their equivalent human-likeness.

The animal-like design offers a further nuance. It achieved the highest warmth ratings of all four designs yet was rated significantly less human-like than both humanoid versions. This pattern is consistent with prior research on zoomorphic robots showing that animal-like designs evoke emotional warmth and perceived approachability through biomorphic cues that operate independently of anthropomorphic resemblance (Shibata and Wada, 2011; Schnitzer et al., 2024; Xiao et al., 2025). Another possible explanation for these findings may be that the animal-like appearance may activate a caregiving schema, positioning the user as the robot's carer (Voysey et al., 2025). Rather than perceiving the robot as a guide, users may be inclined to view a zoomorphic companion as an entity that invites nurturing, protection, or emotional care. This social role may be less congruent with the intended role of a breathing companion, which is expected to actively guide users through relaxation exercises. In contrast, the meditative humanoid appearance may more effectively communicate the competence, calmness, and guidance associated with practices of slow-paced breathing. These findings further support the aesthetic-functional congruence framework proposed in this paper.

### 4.3 Robot designs communicate different affordances

The four design versions can be understood as communicating four distinct affordance profiles, each activating different cognitive schemas and shaping different expectations about the robot's emotional role and social capabilities.

The cartoonish humanoid communicated affordances of playful social interaction and interpersonal presence, as reflected in its highest social presence win counts. Its expressive, face-forward design activated schemas associated with companionship and guided support, consistent with CASA paradigm predictions (Nass et al., 1994). However, its child-like aesthetic register introduced schema incongruity with the therapeutic seriousness of a breathing-support context, likely limiting its perceived competence and trustworthiness relative to the meditative humanoid.

The meditative humanoid communicated affordances of calm, serene and purposeful interaction. Its highest rankings on trustworthiness, competence, breathing companion preference, and overall design preference, across both Likert-scale and pairwise analyses, suggest that its serene aesthetic most effectively communicated aesthetic fit with the intended function. Qualitatively, it was described as “probably the best one” in direct comparisons with other versions. Possible associations with mindfulness and contemplative schemas activated by its Buddhist-inspired visual elements appear to have created a congruence between aesthetic design and functional purpose that the other designs did not achieve to the same degree. Consistent with Goetz et al. (2003), this demonstrates that users prefer robots whose appearance matches the sociability and task seriousness of the interaction.

The animal-like design possibly communicated primarily affective and emotional affordances, such as warmth, softness, and non-threatening companionship, without competence-signaling qualities of the meditative humanoid. This is consistent with prior work on zoomorphic robots in therapeutic contexts (Šabanović et al., 2013; Matheus et al., 2025), and suggests that animal-like designs are particularly suitable when emotional warmth and comfort are the primary design goals, while meditative or mindfulness-themed designs may be preferable when both competence and warmth are required.

The mechanomorphic design communicated affordances associated with technical functionality, industrial efficiency, and machine-like operation, which seems to be entirely incongruent with the emotional accessibility, calmness, and psychological safety expected of a breathing companion robot. The consistently lowest evaluations of the machine-like design across all dimensions, together with qualitative descriptions such as “cold,” “clinical,” and “reminiscent of a Dalek,” indicate that strongly technical visual cues fundamentally conflicted with users' mental models of a wellbeing-oriented companion robot. Especially, the comparison to a Dalek, a fictional cyborg from the British science fiction series “Doctor Who,” which is associated with aggression, hostility, and emotional detachment, is particularly revealing. Rather than evoking feelings of calmness, safety, and care, the design activated associations of threat, dehumanization, and machine dominance. This finding underscores the importance of aesthetic-functional congruence in HRI. In this case, when a robot's visual appearance contradicts its intended therapeutic or supportive function, users may form negative evaluations which undermine trust, and acceptance even before interacting with the robot. From the FAF perspective (Sareh and Loudon, 2024), the mechanomorphic design's form communicated affordances appropriate for a very different functional context, creating extreme schema incongruity that produced consistently negative evaluations of the robot (Meyers-Levy and Tybout, 1989). These findings also support Huang and Moore's (2023) argument that affordance design in social robotics should be context-sensitive rather than based on universal morphological strategies, and extend this perspective by showing that aesthetic register, i.e., the emotional and symbolic tone of a design within a morphological category, is as consequential as the morphological category itself.

### 4.4 Design implications for social robots for breathing

The findings of this study have several relevant implications for the design of wellbeing-oriented social robots. Most notably, the results suggest that maximizing humanoid realism should not be considered the default or universally optimal design strategy for robots intended to support emotional wellbeing, stress reduction, or mindfulness-related activities. The findings suggest that aesthetic design within a morphological category matters as much as the category itself. The two humanoid designs in this study differed not in human-likeness but in emotional tone, i.e., playful vs. serene, and this difference produced systematic divergence on competence and trust. For wellbeing applications, serene, simplified, and composed aesthetics appear to communicate greater functional reliability and therapeutic appropriateness than playful or child-directed aesthetics, even when both activate broadly positive social evaluations. Moreover, biomorphic designs, whether humanoid or zoomorphic, were consistently preferable to mechanomorphic designs in emotionally sensitive interaction contexts. The mechanomorphic design's failure across all dimensions suggests that technical or industrial visual cues are fundamentally incompatible with wellbeing-oriented HRI, regardless of the robot's actual functional capabilities. This finding supports the emerging design principle that emotional accessibility should be a primary goal in wellbeing robot design, not a secondary consideration after functional performance.

Our findings also indicate that successful appearance design in wellbeing-oriented HRI may depend less on maximizing anthropomorphic realism and more on communicating calmness, presence, trust and in consequence psychological safety. In the present study, the meditative humanoid version achieved particularly strong evaluations for trustworthiness, social presence, and behavioral preference, while the animal-like version elicited the highest warmth ratings. Both embodiments appeared to communicate interaction qualities congruent with slow-paced breathing exercises without relying on highly realistic human resemblance. This suggests that users may respond positively to designs that evoke emotional comfort and relational accessibility while avoiding the stronger interpersonal expectations associated with highly humanoid agents.

The results therefore support more context-sensitive approaches to aesthetic designs of social robots. In emotionally sensitive interaction contexts, highly anthropomorphic embodiments may not necessarily provide additional advantages and may even increase risks related to expectation formation. Human-like embodiments can evoke assumptions regarding conversational intelligence, empathy, emotional responsiveness, or social understanding that exceed the robot's actual capabilities. Such expectation mismatches may reduce trust or increase disappointment when interaction behavior does not fully align with the promise of anthropomorphism. More simplified appearances may reduce this expectation gap while still enabling meaningful emotional engagement and social interpretation. The findings further suggest that visual designs for wellbeing-oriented robots should communicate calmness, softness, emotional warmth, and non-threatening interaction qualities which may be particularly beneficial for breathing support, mindfulness, stress reduction, and emotional regulation. In contrast, embodiments emphasizing technicality or industrial functionality may conflict users' expectations of emotional support and relaxation, as reflected in the consistently low evaluations of the mechanomorphic version in this study.

Mobed et al. (2024) argue that robot aesthetics can be analyzed along several continuous design dimensions, including natural-mechanical, familiar-strange, and abstract-realistic and broader visual design dimensions such as angularity-curvature, chaos-unity, and accurate-iconographic, suggesting that robot appearance can be understood as a combination of different design characteristics rather than isolated morphological categories. While Mobed et al. (2024) propose robot aesthetic dimensions as a descriptive framework for analyzing robot appearance, our findings suggest that these aesthetic characteristics cannot be interpreted independently of the robot's intended function. In our study, users evaluated the robot variants according to the degree of congruence between their visual appearance and the expected purpose of a breathing companion. Thus, we argue that aesthetic evaluation in HRI is inherently functional and context dependent. Our argument is that users interpret aesthetic design of robots relative to their intended purpose. We propose aesthetic-functional congruence as a complementary concept describing the degree to which a robot's visual appearance communicates and supports its intended function.

The present findings therefore support emerging perspectives in HRI suggesting that design effectiveness depends on the coherence between appearance, expected behavior, and intended interaction context (Goetz et al., 2003; Huang and Moore, 2023). For breathing-support robots such as Neffy, successful design may thus require balancing anthropomorphic cues with aesthetic-functional congruence rather than maximizing human realism alone.

### 4.5 Gender differences and design implications for social robots for breathing

The gender analyses revealed that robot design version was the dominant predictor of all outcomes, with gender effects largely absent or small. The one exception was a significant interaction between gender and robot design version for behavioral intention [*F*_(3, 303)_ = 3.96, *p* = 0.009], indicating that female and male participants responded differently to specific designs in terms of willingness to use the robot for breathing exercises, even though their social evaluations of warmth, competence, and enjoyment were comparable. This dissociation suggests that affective and social evaluations of robot appearance may be relatively automatic and comparable across genders, consistent with CASA paradigm predictions, whereas behavioral intentions involve more deliberate consideration of personal relevance and anticipated use context (Davis, 1989; Venkatesh et al., 2003). A small but significant main effect of gender on competence [*F*_(1, 101)_ = 4.42, *p* = 0.038], with female participants rating robots as slightly more competent overall, is consistent with prior research showing gender differences in technology perception and evaluation (Rosenberg-Kima and Buchem, 2025), though the absence of a significant gender × design interaction for competence indicates that this effect was not design-specific. The observed gender differences in behavioral intention and the attenuating influence of prior interest in robots should therefore be interpreted cautiously. The present study was not designed to identify the psychological mechanisms underlying these effects. Future research should investigate whether factors such as technology attitudes, prior experience with robots, personality characteristics, perceived usefulness, or gender-related expectations mediate or moderate appearance preferences.

### 4.6 Limitations and future directions

Nevertheless, several limitations should be considered when interpreting the findings:

First, the study relied on image- and video-based evaluations rather than direct embodied interaction with a physical robot. This is a particularly relevant limitation for the breathing companion robot Neffy, where haptic contact and synchronized movement are central to the HRI experience. Perceived warmth, trust, and aesthetic-functional congruence may shift substantially once users place their hands on the robot's shoulders and experience its breathing rhythm in real time. Evaluations based on visual stimuli alone may therefore overestimate or underestimate acceptance outcomes that depend on multimodal and embodied interaction qualities.

Second, participants evaluated AI-generated visual representations rather than fully functional physical prototypes. While the stimulus design was carefully controlled and grounded in the actual interaction context through scene compositing, AI-generated images may not fully capture the material qualities, including texture, weight, warmth, sound, movement smoothness, which are likely to contribute to user perception and acceptance of a physical robot. Future studies should replicate the design comparison using physical prototypes to examine whether the aesthetic-functional congruence effects observed here generalize to embodied interaction.

Third, the study measured anticipated evaluations and behavioral intentions rather than actual use behavior, long-term engagement, or physiological and psychological outcomes during breathing practice. Behavioral intention is a well-validated predictor of technology use (Davis, 1989; Venkatesh et al., 2003), but the relationship between intended and actual use may be moderated by factors not captured in a single-session online evaluation, including habituation, expectation disconfirmation, and the quality of the embodied breathing experience itself.

Fourth, although the four design versions enabled a controlled comparison of broad aesthetic designs, i.e., cartoonish humanoid, animal-like, mechanomorphic, and meditative humanoid, they do not isolate the effects of specific visual features. The higher rating for the meditative humanoid design in competence and trustworthiness may reflect its facial expression, color palette, material softness, simplified proportions, or its Buddhist-inspired visual symbolism, or some combination of these. Future research using systematic feature manipulation, such as varying facial expression, eye shape, color, or cultural symbolism independently, would allow more precise identification of the design features that drive aesthetic-functional congruence in wellbeing contexts.

Fifth, the cultural specificity of the meditative humanoid design warrants explicit acknowledgment. Its golden dome and serene facial expression draw on visual conventions associated with Buddhist and broader East Asian contemplative traditions. While these cues appear to have activated mindfulness schemas among predominantly middle-aged (mean age: *M* = 38.97 years, range: 18–79 years) study participants from Western countries (majority from the USA and UK), their meaning and effectiveness may differ substantially across cultural contexts. Participants from older generations and/or other cultures with different religious or philosophical traditions, or with less familiarity with Buddhist iconography, may interpret the same design cues differently, potentially as foreign, unfamiliar, or even inappropriate, which could reverse the observed results for this version.

Sixth, the sample was recruited via the online platform Prolific and consisted predominantly of English-speaking participants from the United States (*n* = 65) and the United Kingdom (*n* = 31), with only a small number of participants from other countries. This limits the generalizability of the findings in several important ways. Cultural differences in attitudes toward robots, in familiarity with mindfulness and meditation practices, in aesthetic preferences for robot design, and in the schemas activated by humanoid vs. zoomorphic vs. mechanomorphic appearances are well-documented (Kim et al., 2021). Cultures with higher robot acceptance, such as Japan, or with stronger contemplative traditions may respond differently to the meditative humanoid version aesthetic. Cultures where robots are viewed with greater skepticism or where mindfulness schemas are less popular and culturally embedded may show weaker aesthetic-functional congruence effects for this design. Future cross-cultural studies are needed to examine whether the advantage of the meditative humanoid design for a breathing-support robot generalizes beyond Western, English-speaking populations, and to identify which aspects of aesthetic-functional congruence are universal vs. culturally situated.

Seventh, the sample characteristics limit generalizability to specific user populations for which breathing companion robots may be particularly relevant. The study sample was drawn from a general adult population with moderate experience with breathing exercises and meditation, and did not include clinical populations such as individuals with anxiety disorders, chronic pain, or respiratory conditions, which are groups for whom breathing support robots may offer the greatest benefit and who may evaluate design aesthetics through a different lens shaped by personal health experience and therapeutic need. Future research should therefore extend this work to clinical and elderly populations, as well as to children, for whom zoomorphic or cartoonish humanoid designs may be more appropriate than the meditative aesthetic preferred by our general adult sample.

Finally, the present findings should be interpreted within the specific context of a tabletop breathing companion robot supporting individual slow-breathing exercises. Appearance preferences may differ substantially across application domains, such as clinical rehabilitation, workplace stress management, educational settings, or domestic companionship, where users may hold different expectations regarding aesthetic design features of such robots. For example, an animal-like design resembling a toy puppy may be particularly appropriate in domestic companionship or pediatric rehabilitation as it can be more appealing to families and younger users. In elderly care or hospital settings, however, the same design may be perceived as insufficiently professional or “childish,” whereas a more restrained mechanical or clinical design may better convey functional competence and safety. Similarly, in workplace settings, adult users may prefer a discreet and minimalistic appearance that can be integrated into an office environment without seeming overly intimate. In educational settings, students, depending on their age and culture, may prefer visually engaging designs that resemble familiar characters from popular culture. These examples illustrate that aesthetic-functional congruence is likely to depend not only on the robot's task, but also on the settings, age, gender and culture-related factors, social norms and other user characteristics associated with the context of use. Future studies should therefore examine aesthetic-functional congruence across diverse contexts of use, employing physical robot prototypes, culturally diverse participant samples, different user groups, and systematic manipulation of individual appearance variables to develop a more comprehensive theory of robot appearance design.

## 5 Conclusions

This study investigated how different robot embodiments influence social evaluation and anticipated acceptance of Neffy, a breathing-support social robot designed for stress reduction and wellbeing support. The findings demonstrate that perceived human-likeness significantly shaped participants' evaluations across multiple dimensions, including warmth, competence, trustworthiness, social presence, perceived enjoyment, and behavioral intention. Embodiments perceived as more human-like were generally evaluated more positively, supporting previous HRI research suggesting that anthropomorphic cues activate social-cognitive processes associated with human interaction partners.

At the same time, the results clearly indicate that human-like appearance alone was insufficient to explain participants' preferences and acceptance of the robot embodiments. Most notably, the symbolic version achieved evaluations comparable to or exceeding the humanoid version across several dimensions, particularly trustworthiness, social presence, and behavioral preference. Similarly, the animal-like version elicited the highest warmth ratings despite lower perceived human-likeness, yet scored below meditative humanoid version. In contrast, the mechanomorphic version consistently received the lowest evaluations across warmth, trust, enjoyment, and behavioral intention. These findings suggest that participants evaluated different aesthetic robot designs in relation to their functional purpose of a breathing companion robot.

These findings support our concept of aesthetic-functional congruence in HRI. Embodiments perceived as emotionally congruent with breathing exercises, calmness, mindfulness, and emotional support were evaluated more positively than embodiments communicating technicality or industrial functionality. The results further suggest that different embodiments communicate different social and emotional affordances that shape expectations regarding the robot's role, capabilities, and appropriateness for emotionally sensitive interaction contexts.

Our findings should also be interpreted in light of previous work indicating that aesthetic evaluations of robots may change over time, from early impressions to enduring preferences, as users gain experience with a robot (Colle and Dragone, 2026). Furthermore, as aesthetic responses are influenced by multiple factors possibly including age, gender, cultural background, prior knowledge, familiarity with robots (Colle and Dragone, 2026) and interest in robots (as our results show), more research is needed to examine the role of congruence between a robot's aesthetic and functional design for acceptance and sustained engagement over time, especially with companion robots.

From a theoretical perspective, the study extends previous work on anthropomorphism and social robot design by integrating schema congruity theory, affordance theory, and aesthetic-function congruence into the evaluation of wellbeing-oriented robots. The findings suggest that positive evaluations do not necessarily emerge from anthropomorphism or realism altogether, but rather from a coherent balance between aesthetic cues and functional capabilities. In this sense, a symbolic version may represent a form of “appropriate anthropomorphism” (Wang et al., 2025) that supports engagement without evoking overly strong expectations associated with highly humanoid agents.

The findings also have important implications for the design of breathing-support and wellbeing-oriented robots. Rather than maximizing (human or animal) realism, designers may benefit from focusing on embodiments that communicate calmness, emotional warmth, trust, and psychological safety in the first place. More abstract, symbolic, or zoomorphic embodiments may therefore represent promising alternatives to strongly realistic designs in applications related to therapeutic support such anxiety and stress reduction, mindfulness, emotional regulation (Šabanović et al., 2013).

The present study suggests that successful robot design in wellbeing-oriented HRI depends not solely on increasing realism or choosing “the right” morphology, but on creating aesthetically and functionally congruent interaction experiences that align with users' expectations and interpretations of the robot's intended role and task.

## Data Availability

The original contributions presented in the study are included in the article/Supplementary material, further inquiries can be directed to the corresponding author.
