# Supplementary material for: Calm beats cute: designing aesthetic-functional congruence in human-robot interaction for breathing companion robots
Source: Front Cognit. 2026 Sep 23;5:1886795. doi: 10.3389/fcogn.2026.1886795 (PMC13642192; doi:10.3389/fcogn.2026.1886795)
Supplement: Supplementary file 1 [file Data_Sheet_1.pdf]

Calm Beats Cute: Aesthetic Register and Social Evaluation of Four Appearance Designs for a Breathing Companion Robot.

Ilona Buchem[1], Rinat B. Rosenberg-Kima[2*], Emily Evermann[1] , Mandana Sutter[3]

[1]Laboratory or Communication, Department of Economics and Social Sciences, Berlin University of Applied Sciences, Berlin, Germany

[2]The Mindful Learning Technologies Lab, Faculty of Education in Science and Technology, Technion – Israel Institute of Technology, Haifa, Israel

[3]Institute of Psychology and Ergonomics, Faculty of Mechanical Engineering and Transport Systems Faculty, Technical University of Berlin, Berlin Germany

*** Correspondence:**
Ilona Buchem
buchem@bht-berlin.de

Keywords: social robots; human–robot interaction; embodiment; human-likeness; technology acceptance; user experience; breathing companion robot; affordance and schema congruity.

Evaluation criterial for the 10 candidate images (evaluation by the internal research team):

1. Clarity of the intended embodiment category

Definition: The extent to which the candidate can be unambiguously recognized as representing its intended category: cartoonish humanoid, animal-like, machine-like, or meditative humanoid. (Rating scale: 1 = not clear, 5 = very clear).

2. Consistency with the original Neffy morphology

Definition: The extent to which the candidate preserves the defining physical structure and identity of the original Neffy robot despite changes in visual style. (Rating scale: 1 = Not consistent; 5 = Very consistent).

3. Aesthetic coherence

Definition: The extent to which the visual elements form a unified and internally consistent design corresponding to the intended aesthetic concept. (Rating scale: 1 = Not coherent; 5 = Very coherent)

4. Preservation of the intended interaction affordances

Definition: The extent to which the design visually and physically communicates that a user can place both hands on the robot's upper body or shoulders and follow its breathing movement (Rating scale: 1 = Not preserved; 5 = Fully preserved).

5. Suitability for a breathing companion application

Definition: The extent to which the appearance seems appropriate for a robot intended to support slow-paced breathing, relaxation, and stress reduction (Rating scale: 1 = Not suitable; 5 = Highly suitable).

Supplementary Table S3. Descriptive statistics (Mean and Standard Deviation) for all 10 image variants evaluated by four expert raters (N = 4).

Image Variant	Overall M (SD)	Clarity M (SD)	Consistency M (SD)	Coherence M (SD)	Affordances M (SD)	Suitability M (SD)
**1**	**4.35 (0.72)**	5.00 (0.00)	4.50 (1.00)	5.00 (0.00)	4.00 (1.41)	3.25 (1.50)
**2**	3.50 (0.82)	3.50 (0.58)	4.25 (0.96)	3.25 (0.50)	3.50 (1.73)	3.00 (0.82)
**3**	**4.35 (1.05)**	5.00 (0.00)	4.00 (2.00)	5.00 (0.00)	3.75 (1.89)	4.00 (1.41)
**4**	3.35 (0.38)	4.25 (0.96)	3.00 (1.41)	4.00 (0.82)	3.25 (1.50)	2.25 (0.50)
**5**	**4.00 (1.51)**	4.25 (0.96)	4.00 (2.00)	4.00 (1.41)	4.00 (1.41)	3.75 (1.89)
**6**	2.75 (0.77)	3.00 (0.82)	2.50 (1.00)	3.25 (0.50)	3.00 (1.83)	2.00 (1.41)
**7**	2.80 (0.71)	2.50 (1.29)	3.50 (0.58)	2.25 (0.96)	3.50 (1.73)	2.25 (0.50)
**8**	2.55 (0.84)	3.00 (1.41)	3.00 (1.41)	2.75 (1.71)	3.00 (1.16)	1.00 (0.00)
**9**	**4.70 (0.35)**	4.75 (0.50)	5.00 (0.00)	4.75 (0.50)	4.25 (0.50)	4.75 (0.50)
**10**	3.20 (0.67)	3.00 (0.82)	3.50 (0.58)	3.25 (1.26)	3.25 (0.96)	3.00 (0.82)

Note. Values are presented as Mean (Standard Deviation). Ratings were provided by four expert raters on a 5-point Likert scale, with higher scores indicating more positive evaluations

Preview mode

Published

Copy responder link

Internal rating Neffy - variant 10

* Indicates required question

← Preview mode Published Copy responder link

as representing its intended category: cartoonish humanoid, animal-like, machine-like, or meditative humanoid.

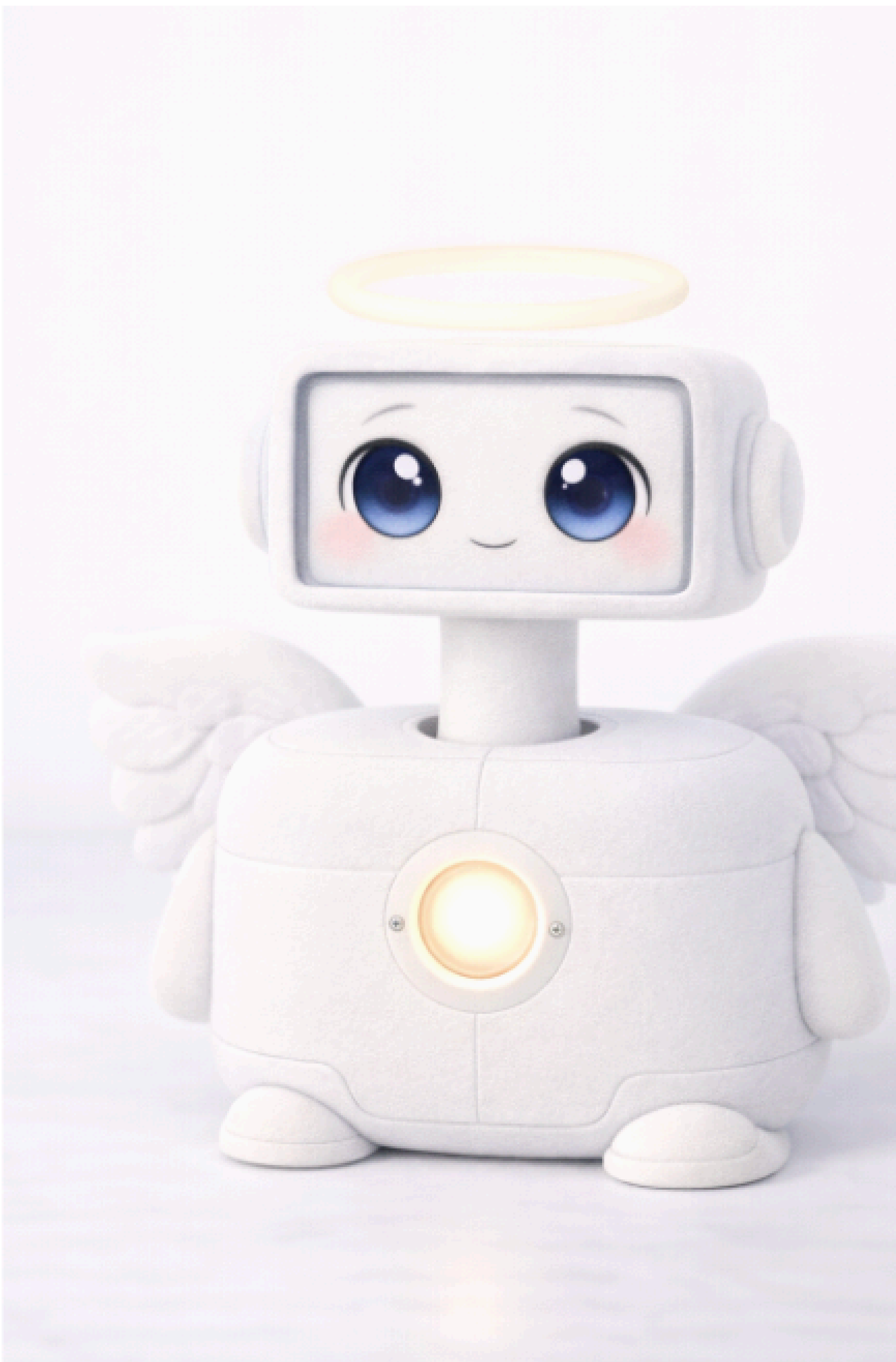

	1	2	3	4	5	
Not clear	○	○	○	○	○	Very clear

Preview mode Published Copy responder link

structure and identity of the original Neffy robot despite changes in visual style.

	1	2	3	4	5	
Not consistent	○	○	○	○	○	Very consistent

3. Aesthetic coherence *
Definition: The extent to which the visual elements form a unified and internally consistent design corresponding to the intended aesthetic concept.

	1	2	3	4	5	
Not coherent	○	○	○	○	○	Very coherent

4. Preservation of the intended interaction affordances *
Definition: The extent to which the design visually and physically communicates that a user can place both hands on the robot's upper body or shoulders and follow its breathing movement.

	1	2	3	4	5	
Not preserved	○	○	○	○	○	Fully preserved

5. Suitability for a breathing companion application *
Definition: The extent to which the appearance seems appropriate for a robot intended to support slow-paced breathing, relaxation, and stress reduction.

	1	2	3	4	5	
Not suitable	○	○	○	○	○	Highly suitable

Preview mode

Published

Copy responder link

This content is neither created nor endorsed by Google. - Contact form owner - Terms of Service - Privacy Policy

Does this form look suspicious? Report

Google Forms

Preview mode

Published

Copy responder link

Preview mode

Published

Copy responder link

Internal rating Neffy - variant 9

* Indicates required question

Preview mode Published Copy responder link

as representing its intended category: cartoonish humanoid, animal-like, machine-like, or meditative humanoid.

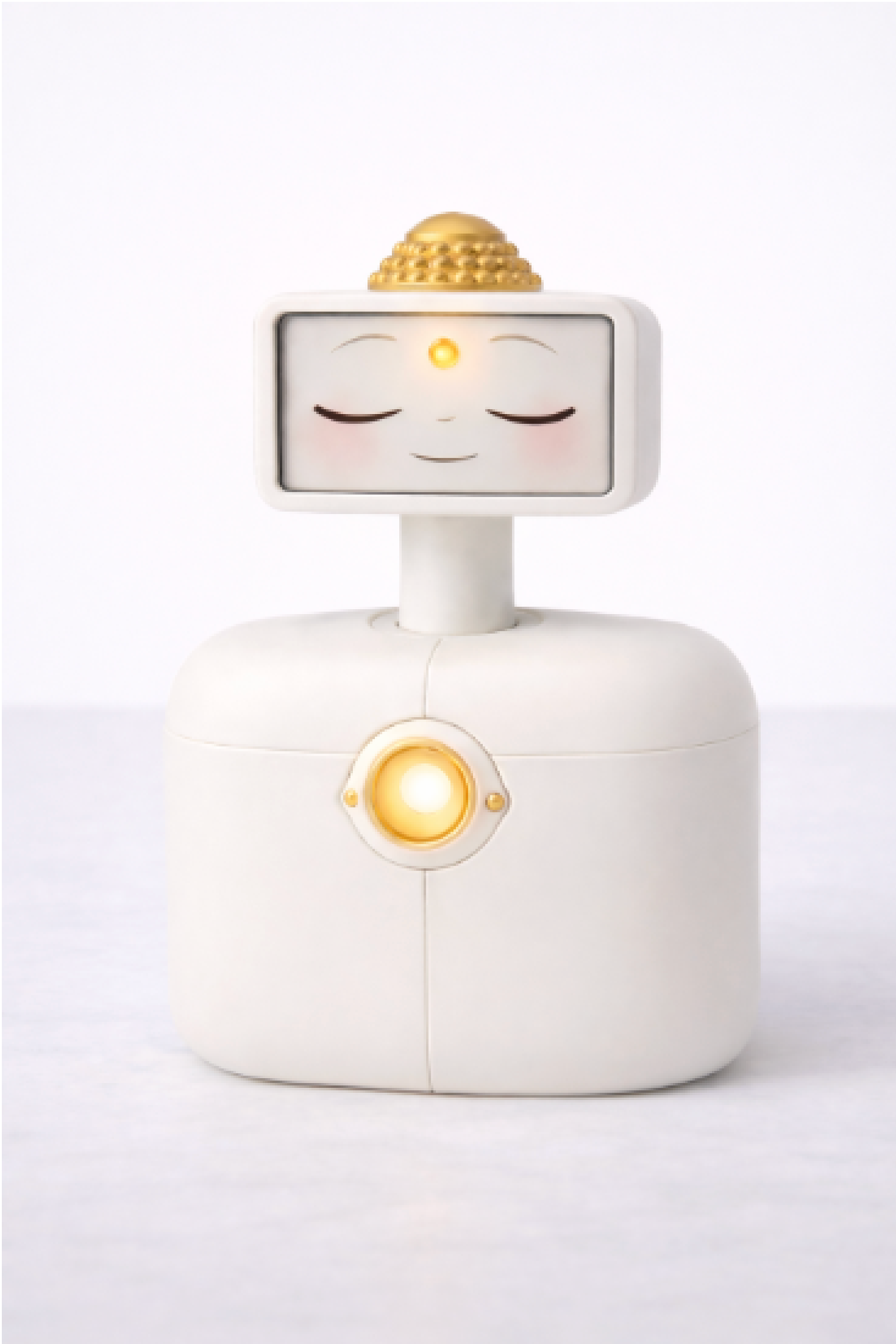

	1	2	3	4	5	
Not clear	○	○	○	○	○	Very clear

Preview mode Published Copy responder link

structure and identity of the original Neffy robot despite changes in visual style.

	1	2	3	4	5	
Not consistent	○	○	○	○	○	Very consistent

3. Aesthetic coherence *
Definition: The extent to which the visual elements form a unified and internally consistent design corresponding to the intended aesthetic concept.

	1	2	3	4	5	
Not coherent	○	○	○	○	○	Very coherent

4. Preservation of the intended interaction affordances *
Definition: The extent to which the design visually and physically communicates that a user can place both hands on the robot's upper body or shoulders and follow its breathing movement.

	1	2	3	4	5	
Not preserved	○	○	○	○	○	Fully preserved

5. Suitability for a breathing companion application *
Definition: The extent to which the appearance seems appropriate for a robot intended to support slow-paced breathing, relaxation, and stress reduction.

	1	2	3	4	5	
Not suitable	○	○	○	○	○	Highly suitable

Preview mode

Published

Copy responder link

This content is neither created nor endorsed by Google. - Contact form owner - Terms of Service - Privacy Policy

Does this form look suspicious? Report

Google Forms

Preview mode

Published

Copy responder link

Preview mode

Published

Copy responder link

Internal rating Neffy - variant 8

* Indicates required question

← Preview mode Published Copy responder link

as representing its intended category: cartoonish humanoid, animal-like, machine-like, or meditative humanoid.

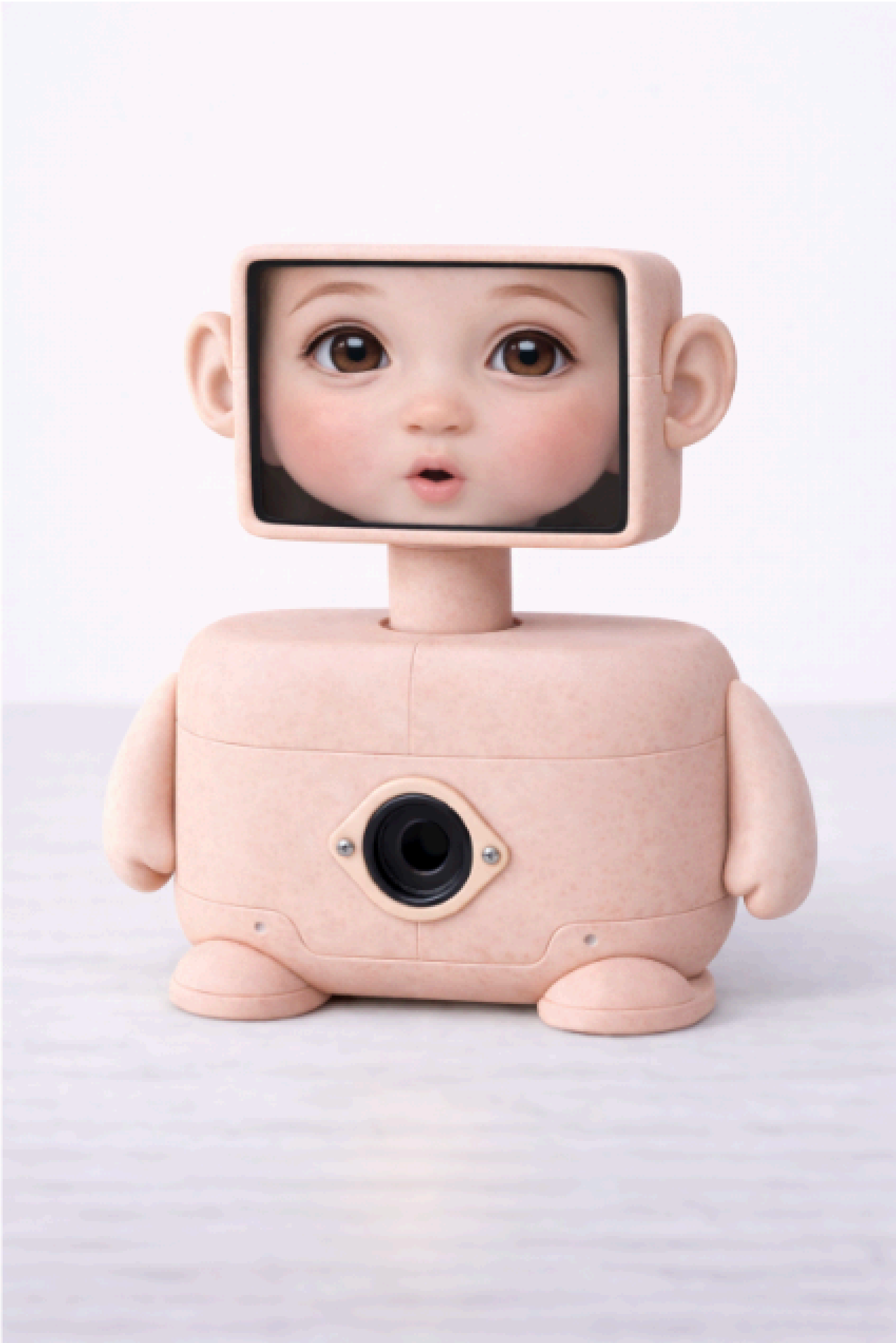

	1	2	3	4	5	
Not clear	○	○	○	○	○	Very clear

Preview mode Published Copy responder link

structure and identity of the original Neffy robot despite changes in visual style.

	1	2	3	4	5	
Not consistent	○	○	○	○	○	Very consistent

3. Aesthetic coherence *

Definition: The extent to which the visual elements form a unified and internally consistent design corresponding to the intended aesthetic concept.

	1	2	3	4	5	
Not coherent	○	○	○	○	○	Very coherent

4. Preservation of the intended interaction affordances *

Definition: The extent to which the design visually and physically communicates that a user can place both hands on the robot's upper body or shoulders and follow its breathing movement.

	1	2	3	4	5	
Not preserved	○	○	○	○	○	Fully preserved

5. Suitability for a breathing companion application *

Definition: The extent to which the appearance seems appropriate for a robot intended to support slow-paced breathing, relaxation, and stress reduction.

	1	2	3	4	5	
Not suitable	○	○	○	○	○	Highly suitable

Preview mode

Published

Copy responder link

This content is neither created nor endorsed by Google. - Contact form owner - Terms of Service - Privacy Policy

Does this form look suspicious? Report

Google Forms

← Preview mode

Published

Copy responder link

Preview mode

Published

Copy responder link

Internal rating Neffy - variant 7

* Indicates required question

← Preview mode Published Copy responder link

as representing its intended category: cartoonish humanoid, animal-like, machine-like, or meditative humanoid.

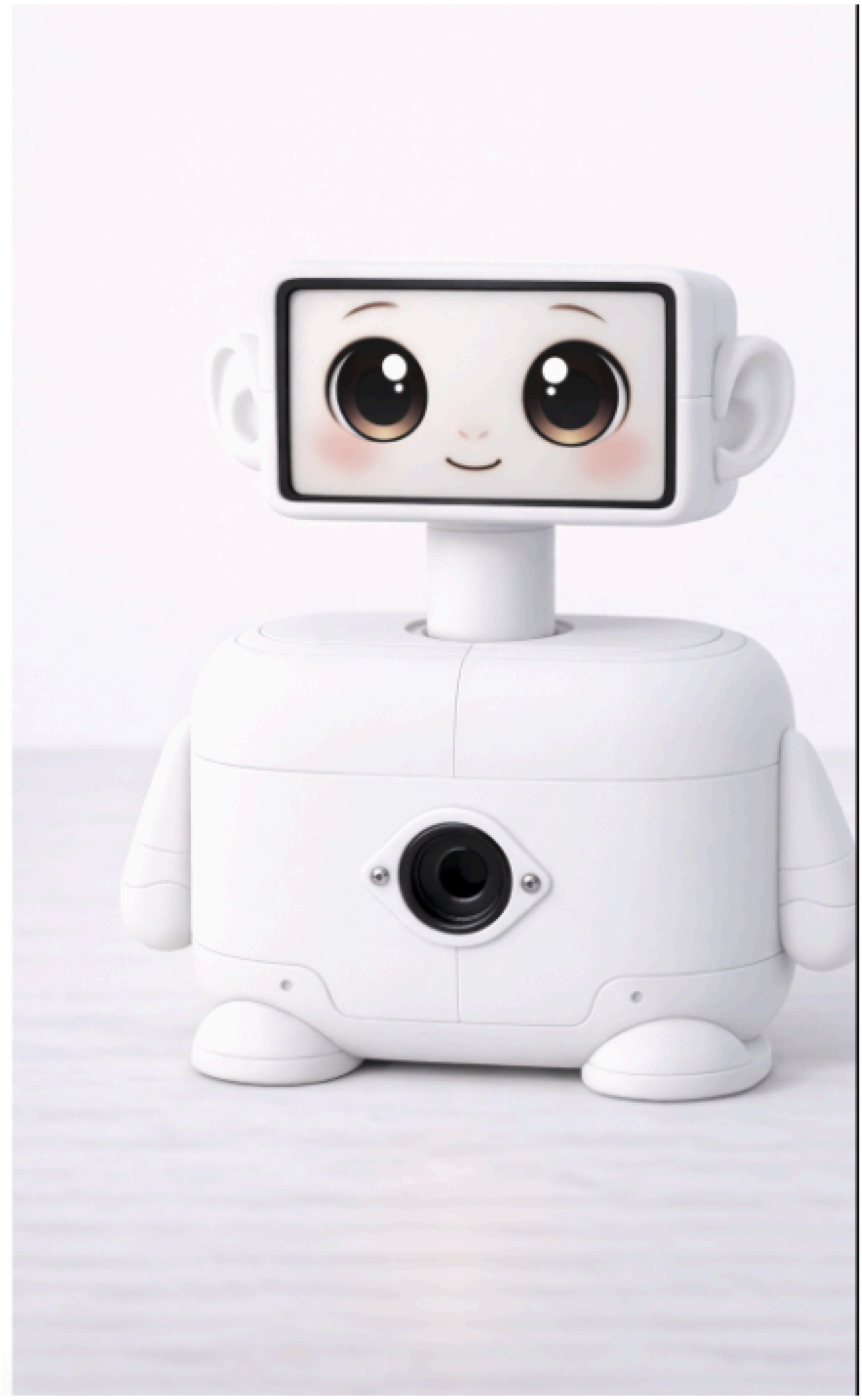

	1	2	3	4	5	
Not clear	○	○	○	○	○	Very clear

Preview mode Published Copy responder link

structure and identity of the original Neffy robot despite changes in visual style.

	1	2	3	4	5	
Not consistent	○	○	○	○	○	Very consistent

3. Aesthetic coherence *
Definition: The extent to which the visual elements form a unified and internally consistent design corresponding to the intended aesthetic concept.

	1	2	3	4	5	
Not coherent	○	○	○	○	○	Very coherent

4. Preservation of the intended interaction affordances *
Definition: The extent to which the design visually and physically communicates that a user can place both hands on the robot's upper body or shoulders and follow its breathing movement.

	1	2	3	4	5	
Not preserved	○	○	○	○	○	Fully preserved

5. Suitability for a breathing companion application *
Definition: The extent to which the appearance seems appropriate for a robot intended to support slow-paced breathing, relaxation, and stress reduction.

	1	2	3	4	5	
Not suitable	○	○	○	○	○	Highly suitable

Preview mode

Published

Copy responder link

This content is neither created nor endorsed by Google. - Contact form owner - Terms of Service - Privacy Policy

Does this form look suspicious? Report

Google Forms

Preview mode

Published

Copy responder link

Preview mode

Published

Copy responder link

Internal rating Neffy - variant 6

* Indicates required question

Preview mode

Published

Copy responder link

as representing its intended category: cartoonish humanoid, animal-like, machine-like, or meditative humanoid.

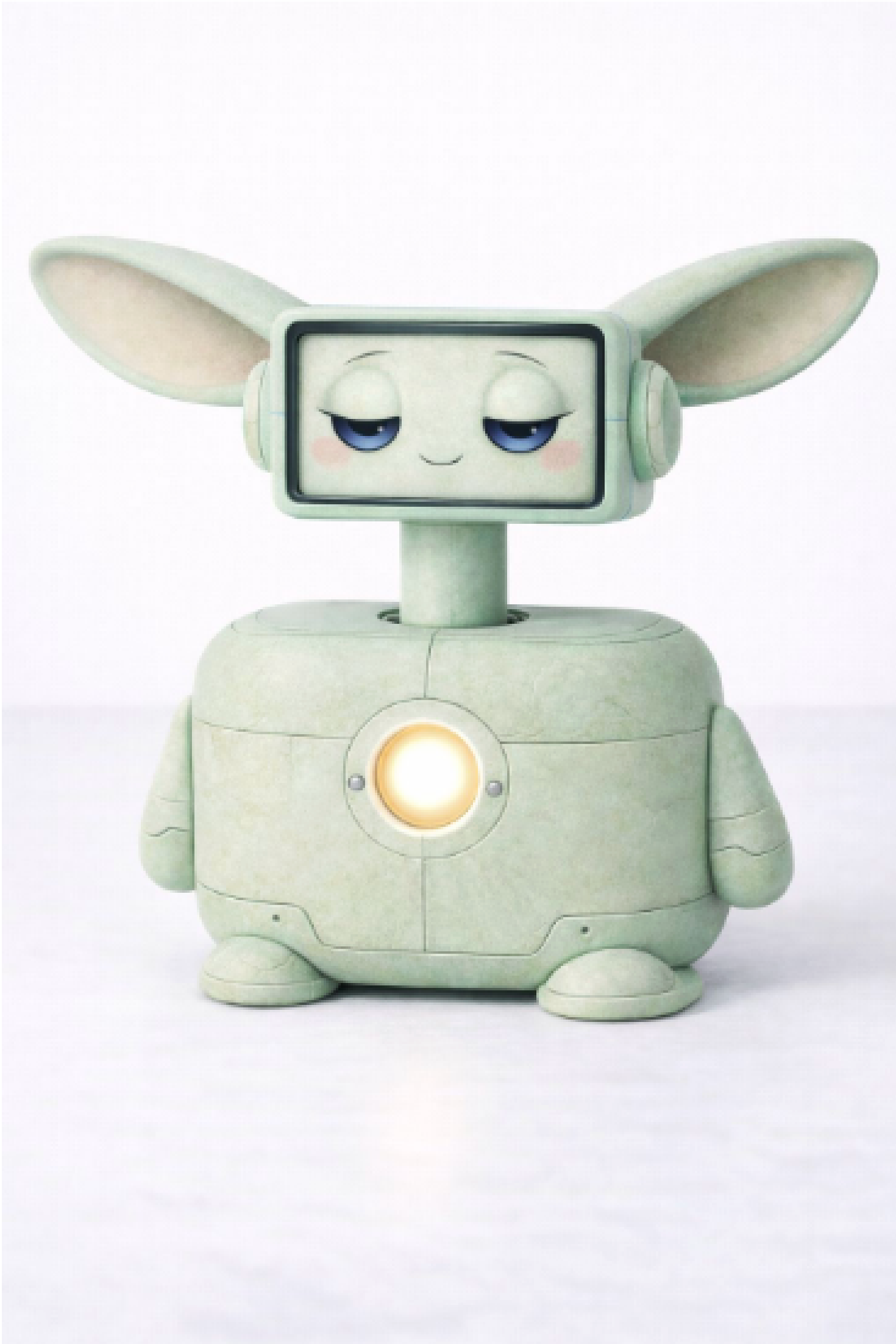

	1	2	3	4	5	
Not clear	○	○	○	○	○	Very clear

Preview mode

Published

Copy responder link

structure and identity of the original Neffy robot despite changes in visual style.

	1	2	3	4	5	
Not consistent	○	○	○	○	○	Very consistent

3. Aesthetic coherence *

Definition: The extent to which the visual elements form a unified and internally consistent design corresponding to the intended aesthetic concept.

	1	2	3	4	5	
Not coherent	○	○	○	○	○	Very coherent

4. Preservation of the intended interaction affordances *

Definition: The extent to which the design visually and physically communicates that a user can place both hands on the robot's upper body or shoulders and follow its breathing movement.

	1	2	3	4	5	
Not preserved	○	○	○	○	○	Fully preserved

5. Suitability for a breathing companion application *

Definition: The extent to which the appearance seems appropriate for a robot intended to support slow-paced breathing, relaxation, and stress reduction.

	1	2	3	4	5	
Not suitable	○	○	○	○	○	Highly suitable

Preview mode Published Copy responder link

This content is neither created nor endorsed by Google. - Contact form owner - Terms of Service - Privacy Policy

Does this form look suspicious? Report

Google Forms

Preview mode

Published

Copy responder link

Preview mode

Published

Copy responder link

Internal rating Neffy - variant 5

* Indicates required question

← Preview mode

Published

Copy responder link

as representing its intended category: cartoonish humanoid, animal-like, machine-like, or meditative humanoid.

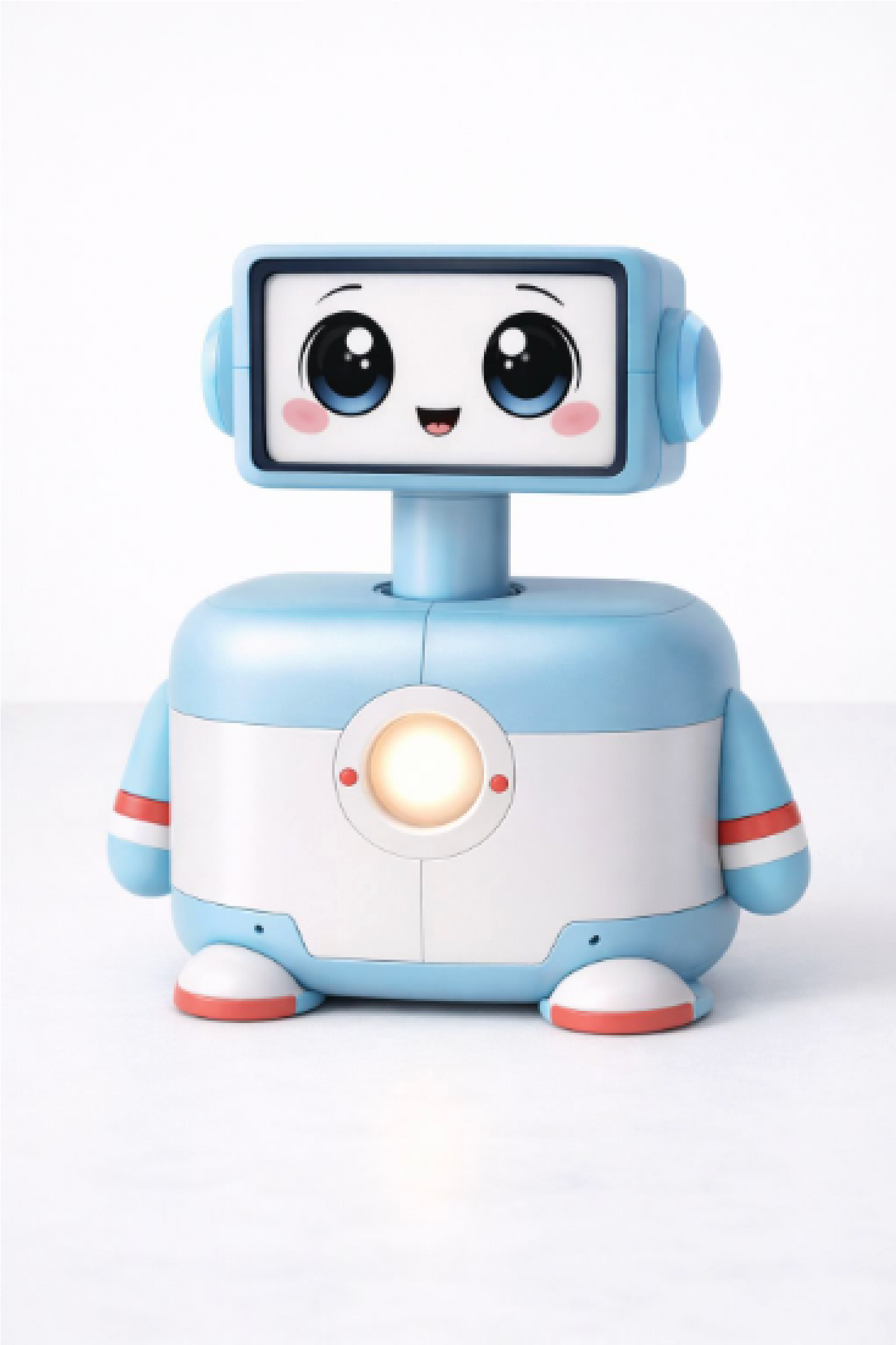

	1	2	3	4	5	
Not clear	○	○	○	○	○	Very clear

Preview mode Published Copy responder link

structure and identity of the original Neffy robot despite changes in visual style.

	1	2	3	4	5	
Not consistent	○	○	○	○	○	Very consistent

3. Aesthetic coherence *

Definition: The extent to which the visual elements form a unified and internally consistent design corresponding to the intended aesthetic concept.

	1	2	3	4	5	
Not coherent	○	○	○	○	○	Very coherent

4. Preservation of the intended interaction affordances *

Definition: The extent to which the design visually and physically communicates that a user can place both hands on the robot's upper body or shoulders and follow its breathing movement.

	1	2	3	4	5	
Not preserved	○	○	○	○	○	Fully preserved

5. Suitability for a breathing companion application *

Definition: The extent to which the appearance seems appropriate for a robot intended to support slow-paced breathing, relaxation, and stress reduction.

	1	2	3	4	5	
Not suitable	○	○	○	○	○	Highly suitable

Preview mode

Published

Copy responder link

This content is neither created nor endorsed by Google. - Contact form owner - Terms of Service - Privacy Policy

Does this form look suspicious? Report

Google Forms

Preview mode

Published

Copy responder link

Preview mode

Published

Copy responder link

Internal rating Neffy - variant 4

* Indicates required question

Preview mode Published Copy responder link

as representing its intended category: cartoonish humanoid, animal-like, machine-like, or meditative humanoid.

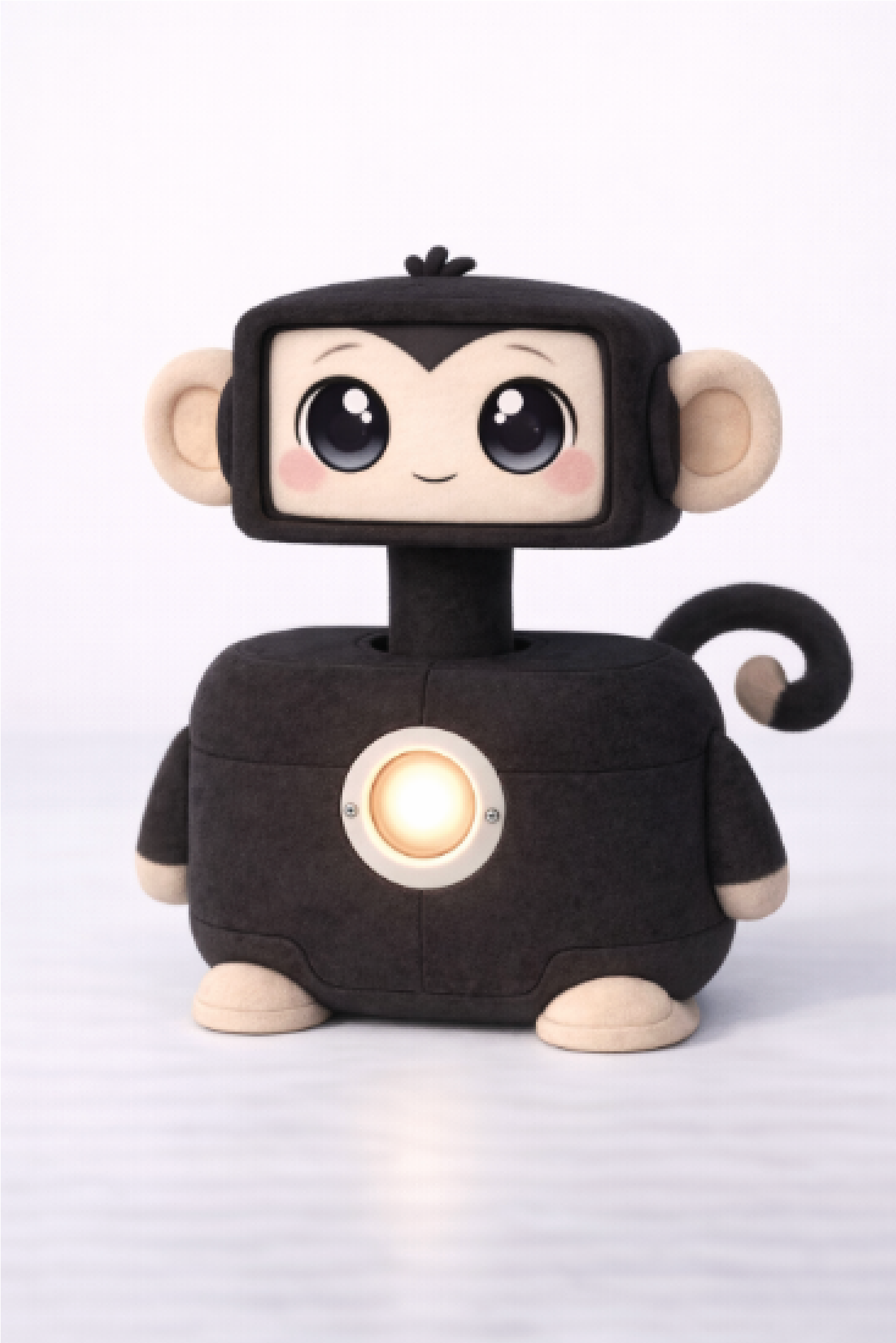

	1	2	3	4	5	
Not clear	○	○	○	○	○	Very clear

Preview mode Published Copy responder link

structure and identity of the original Neffy robot despite changes in visual style.

	1	2	3	4	5	
Not consistent	○	○	○	○	○	Very consistent

3. Aesthetic coherence *

Definition: The extent to which the visual elements form a unified and internally consistent design corresponding to the intended aesthetic concept.

	1	2	3	4	5	
Not coherent	○	○	○	○	○	Very coherent

4. Preservation of the intended interaction affordances *

Definition: The extent to which the design visually and physically communicates that a user can place both hands on the robot's upper body or shoulders and follow its breathing movement.

	1	2	3	4	5	
Not preserved	○	○	○	○	○	Fully preserved

5. Suitability for a breathing companion application *

Definition: The extent to which the appearance seems appropriate for a robot intended to support slow-paced breathing, relaxation, and stress reduction.

	1	2	3	4	5	
Not suitable	○	○	○	○	○	Highly suitable

Preview mode

Published

Copy responder link

This content is neither created nor endorsed by Google. - Contact form owner - Terms of Service - Privacy Policy

Does this form look suspicious? Report

Google Forms

Preview mode

Published

Copy responder link

Preview mode

Published

Copy responder link

Internal rating Neffy - variant 3

* Indicates required question

Preview mode

Published

Copy responder link

as representing its intended category: cartoonish humanoid, animal-like, machine-like, or meditative humanoid.

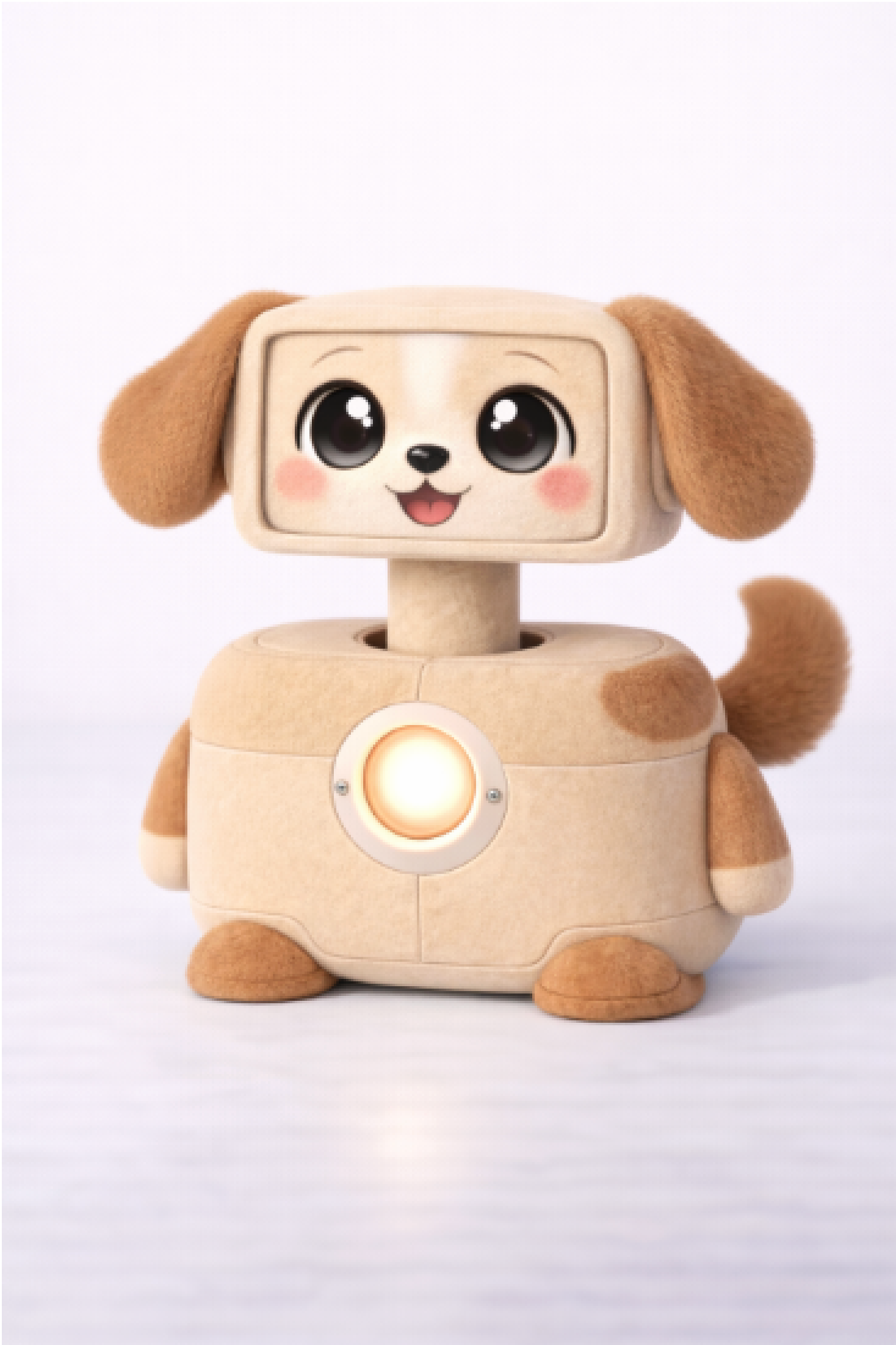

	1	2	3	4	5	
Not clear	○	○	○	○	○	Very clear

Preview mode Published Copy responder link

structure and identity of the original Neffy robot despite changes in visual style.

	1	2	3	4	5	
Not consistent	○	○	○	○	○	Very consistent

3. Aesthetic coherence *

Definition: The extent to which the visual elements form a unified and internally consistent design corresponding to the intended aesthetic concept.

	1	2	3	4	5	
Not coherent	○	○	○	○	○	Very coherent

4. Preservation of the intended interaction affordances *

Definition: The extent to which the design visually and physically communicates that a user can place both hands on the robot's upper body or shoulders and follow its breathing movement.

	1	2	3	4	5	
Not preserved	○	○	○	○	○	Fully preserved

5. Suitability for a breathing companion application *

Definition: The extent to which the appearance seems appropriate for a robot intended to support slow-paced breathing, relaxation, and stress reduction.

	1	2	3	4	5	
Not suitable	○	○	○	○	○	Highly suitable

Preview mode

Published

Copy responder link

This content is neither created nor endorsed by Google. - Contact form owner - Terms of Service - Privacy Policy

Does this form look suspicious? Report

Google Forms

Preview mode

Published

Copy responder link

Preview mode

Published

Copy responder link

Internal rating Neffy - variant 2

* Indicates required question

Preview mode Published Copy responder link

as representing its intended category: cartoonish humanoid, animal-like, machine-like, or meditative humanoid.

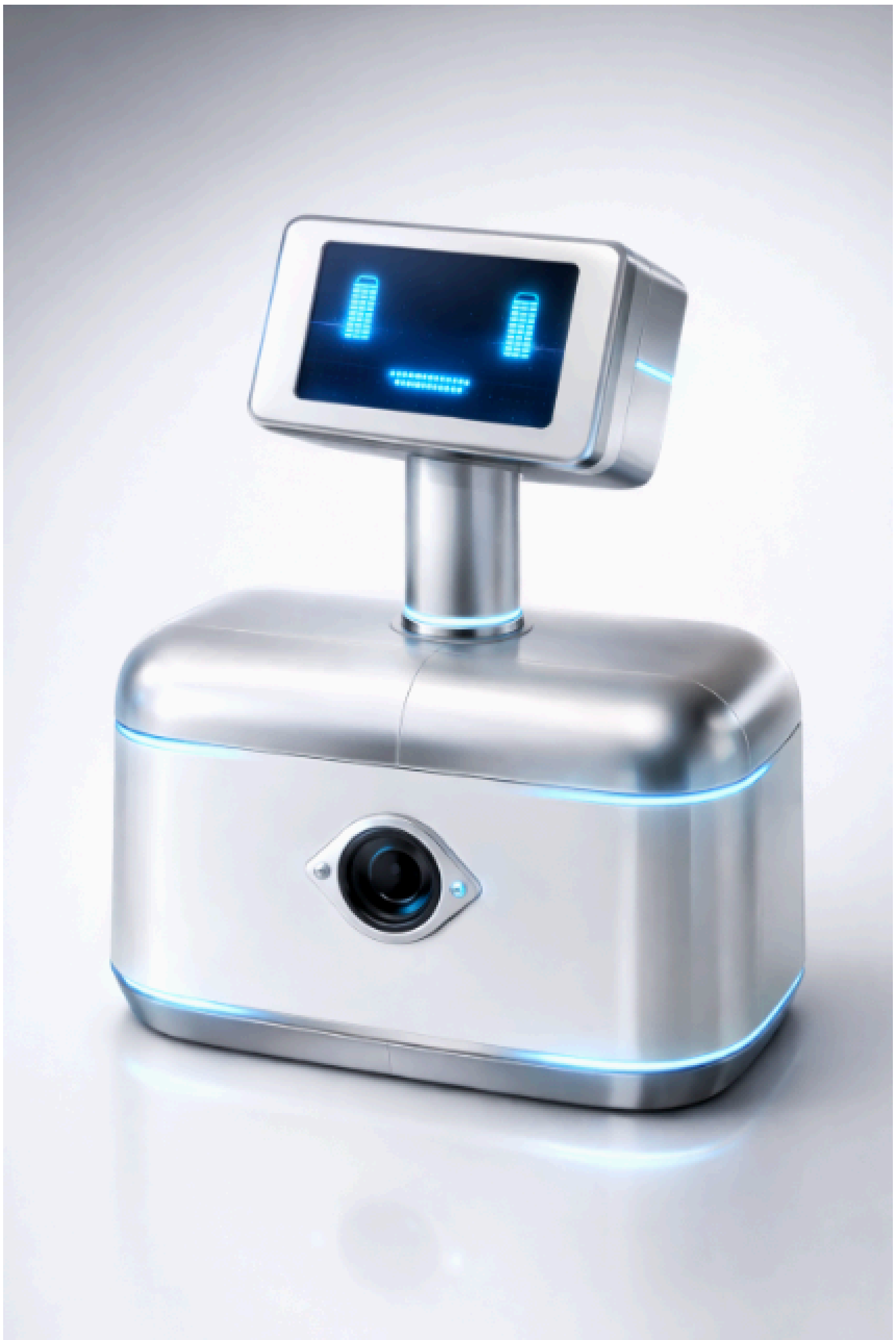

	1	2	3	4	5	
Not clear	○	○	○	○	○	Very clear

Preview mode Published Copy responder link

structure and identity of the original Neffy robot despite changes in visual style.

	1	2	3	4	5	
Not consistent	○	○	○	○	○	Very consistent

3. Aesthetic coherence *

Definition: The extent to which the visual elements form a unified and internally consistent design corresponding to the intended aesthetic concept.

	1	2	3	4	5	
Not coherent	○	○	○	○	○	Very coherent

4. Preservation of the intended interaction affordances *

Definition: The extent to which the design visually and physically communicates that a user can place both hands on the robot's upper body or shoulders and follow its breathing movement.

	1	2	3	4	5	
Not preserved	○	○	○	○	○	Fully preserved

5. Suitability for a breathing companion application *

Definition: The extent to which the appearance seems appropriate for a robot intended to support slow-paced breathing, relaxation, and stress reduction.

	1	2	3	4	5	
Not suitable	○	○	○	○	○	Highly suitable

Preview mode

Published

Copy responder link

This content is neither created nor endorsed by Google. - Contact form owner - Terms of Service - Privacy Policy

Does this form look suspicious? Report

Google Forms

Preview mode

Published

Copy responder link

Preview mode Published Copy responder link

Internal rating Neffy - variant 1

* Indicates required question

Preview mode Published Copy responder link

as representing its intended category: cartoonish humanoid, animal-like, machine-like, or meditative humanoid.

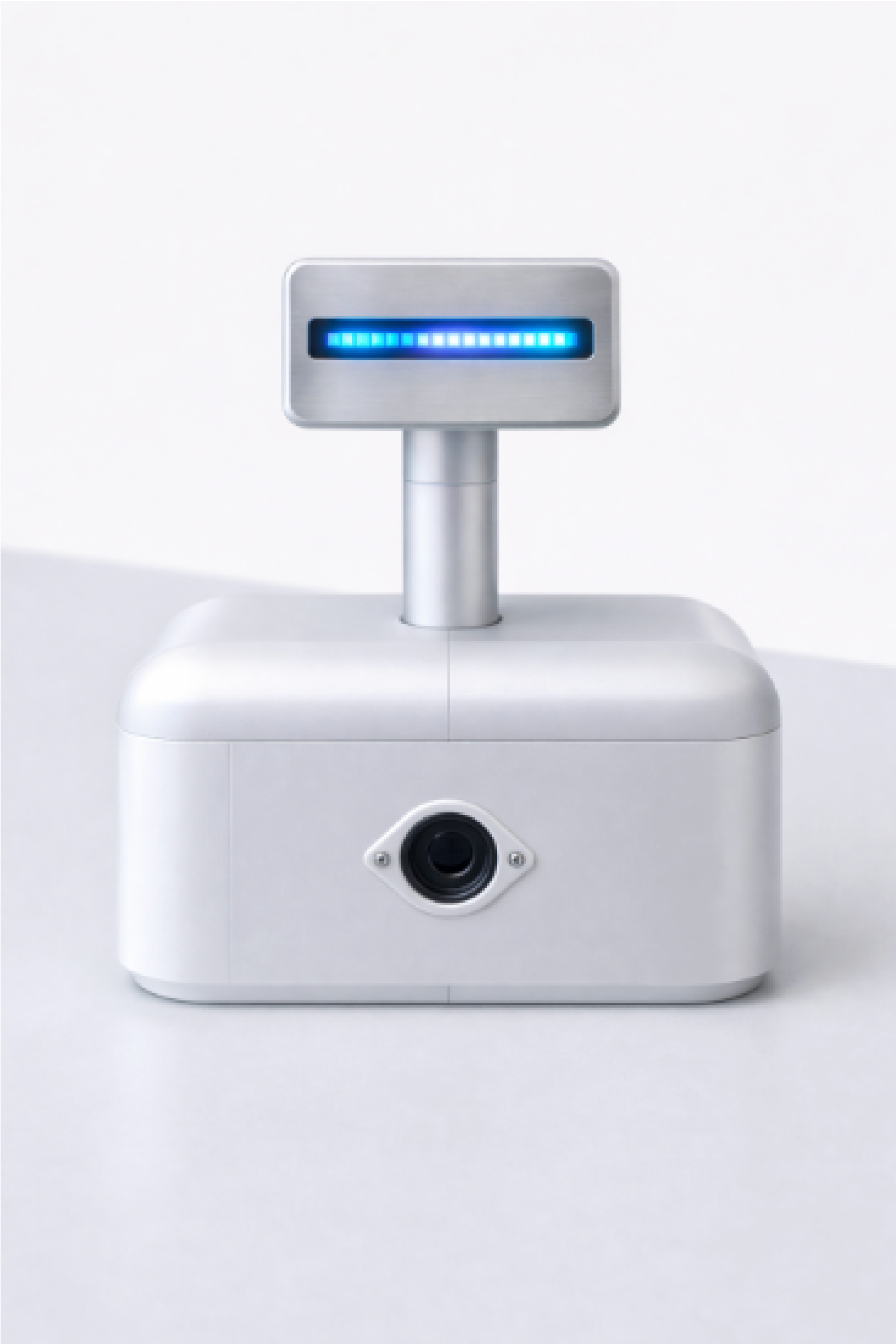

	1	2	3	4	5	
Not clear	○	○	○	○	○	Very clear

Preview mode

Published

Copy responder link

structure and identity of the original Neffy robot despite changes in visual style.

	1	2	3	4	5	
Not consistent	○	○	○	○	○	Very consistent

3. Aesthetic coherence *

Definition: The extent to which the visual elements form a unified and internally consistent design corresponding to the intended aesthetic concept.

	1	2	3	4	5	
Not coherent	○	○	○	○	○	Very coherent

4. Preservation of the intended interaction affordances *

Definition: The extent to which the design visually and physically communicates that a user can place both hands on the robot's upper body or shoulders and follow its breathing movement.

	1	2	3	4	5	
Not preserved	○	○	○	○	○	Fully preserved

5. Suitability for a breathing companion application *

Definition: The extent to which the appearance seems appropriate for a robot intended to support slow-paced breathing, relaxation, and stress reduction.

	1	2	3	4	5	
Not suitable	○	○	○	○	○	Highly suitable

Preview mode

Published

Copy responder link

This content is neither created nor endorsed by Google. - Contact form owner - Terms of Service - Privacy Policy

Does this form look suspicious? Report

Google Forms

Preview mode

Published

Copy responder link
