# Supplementary material for: Calm beats cute: designing aesthetic-functional congruence in human-robot interaction for breathing companion robots
Source: Front Cognit. 2026 Sep 23;5:1886795. doi: 10.3389/fcogn.2026.1886795 (PMC13642192; doi:10.3389/fcogn.2026.1886795)
Supplement: Supplementary file 2 [file Data_Sheet_2.pdf]

Calm Beats Cute: Designing Aesthetic-Functional Congruence in Human-Robot Interaction for Breathing Companion Robots.

Ilona Buchem[1], Rinat B. Rosenberg-Kima[2*], Emily Evermann[1] , Mandana Sutter[3]

[1]Laboratory or Communication, Department of Economics and Social Sciences, Berlin University of Applied Sciences, Berlin, Germany

[2]The Mindful Learning Technologies Lab, Faculty of Education in Science and Technology, Technion – Israel Institute of Technology, Haifa, Israel

[3]Institute of Psychology and Ergonomics, Faculty of Mechanical Engineering and Transport Systems Faculty, Technical University of Berlin, Berlin Germany

*** Correspondence:**
Ilona Buchem
buchem@bht-berlin.de

Keywords: aesthetics; social robots; human–robot interaction; embodiment; human-likeness; technology acceptance; user experience; breathing companion robot.

Supplement 2 Prompts

PROMPT STRUCTURE: 1. General description; 2. Material and Color; 3. Facial Features; 4. Body Details; 5. Movement and Interaction; 6. Design Style 7. Effect and Impression; 8. Rendering.

General Prompt (the same for all images)

This is an image of the Neffy robot. Create a new image of the Neffy robot with the following details: Neffy should be placed on a light grey table and the background should be white. There should be no other details.

1. Neffy as a puppy (animal cub)

Zoomorph – High Familiarity

Prompt: This is an image of the Neffy breathing robot. Create a new image of the Neffy robot with the following details: A small breathing companion robot designed to resemble a friendly puppy. Soft microfiber fur in light beige or golden tones, friendly warm color palette. Large round eyes with large black irises, small upturned mouth. Floppy ears, short gently moving tail. Slow breathing animation and subtle purring sound evoke calmness and warmth. Slightly stylized rather than hyper-realistic to avoid uncanny impressions. Gentle motion and expressive gaze simulate liveliness and trust. Studio lighting, 3D render with tactile realism and highly detailed, high perceived warmth and trust, cute but realistic.

- General: A small breathing companion robot named Neffy designed to resemble a friendly puppy.
- Material & Color: Soft microfiber fur in light beige or golden tones, friendly warm color palette.
- Facial Features: Large round eyes with large black irises, small upturned mouth.
- Body Details: Floppy ears, short gently moving tail.

- Movement & Interaction: Slow breathing animation and subtle purring sound evoke calmness and warmth.
- Design Style: Slightly stylized rather than hyper-realistic to avoid uncanny impressions.
- Effect / Impression: Gentle motion and expressive gaze simulate liveliness and trust.
- Rendering: Studio lighting, 3D render with tactile realism and highly detailed, high perceived warmth and trust, cute but realistic.

2. Neffy as a Machine (Non-Organic Form)

Mechanomorph – Minimalistic Functional

Prompt: A small breathing robot with a sleek mechanical form and no organic traits, no fur or covering. Smooth polymer or brushed metal; cool silver, white, or muted blue tones. No traditional face; a single circular LED symbolizes breathing. Clean geometric silhouette; seamless joints; compact proportions. Soft LED pulsing and subtle rhythmic body expansion indicate breathing. Minimalistic, futuristic, functional; precise and calm visual language. Technologically reliable, serene, slightly distant but trustworthy. Cool-toned lighting; polished futuristic 3D render; reflections emphasize smoothness.

- General: A small breathing robot with a sleek mechanical form and no organic traits, no fur or covering.
- Material & Color: Smooth polymer or brushed metal; cool silver, white, or muted blue tones.
- Facial Features: No traditional face; a single circular LED symbolizes breathing.
- Body Details: Clean geometric silhouette; seamless joints; compact proportions.
- Movement & Interaction: Soft LED pulsing and subtle rhythmic body expansion indicate breathing.
- Design Style: Minimalistic, futuristic, functional; precise and calm visual language.
- Effect / Impression: Technologically reliable, serene, slightly distant but trustworthy.
- Rendering: Cool-toned lighting; polished futuristic 3D render; reflections emphasize smoothness.

3. Neffy as a Cartoon Character

Iconic Anthropomorph – Stylized Friendly

Prompt: A cartoon-inspired companion robot emphasizing exaggerated friendliness. Smooth plastic finish; bright cheerful colors (mainly blue, white, red accents). Extremely simplified face with two very large expressive comic-style eyes; minimal mouth; soft rounded head. Simplified cylindrical body and limbs; minimal robotic detailing. Facial expression dynamically animated (blinking, micro-movements); energetic yet comforting gestures. Flat-shaded or cel-shaded 3D aesthetic and highly stylized cartoon aesthetic; strong outlines and simplified geometry. Friendly, playful, cute, socially engaging; highly approachable and safe for all ages. Bright soft lighting; cel-shaded 3D style; high clarity with soft shadows.

- General: A cartoon-inspired companion robot emphasizing exaggerated friendliness.
- Material & Color: Smooth plastic finish; bright cheerful colors (mainly blue, white, red accents).
- Facial Features: Extremely simplified face with two very large expressive comic-style eyes; minimal mouth; soft rounded head.
- Body Details: Simplified cylindrical body and limbs; minimal robotic detailing.

- Movement & Interaction: Facial expression dynamically animated (blinking, micro-movements); energetic yet comforting gestures.
- Design Style: Flat-shaded or cel-shaded 3D aesthetic and highly stylized cartoon aesthetic; strong outlines and simplified geometry.
- Effect / Impression: Friendly, playful, cute, socially engaging; highly approachable and safe for all ages.
- Rendering: Bright soft lighting; cel-shaded 3D style; high clarity with soft shadows.

4. Neffy as a Meditative Buddha-Like Figure

Serene Anthropomorph – Calm Spiritual Companion

Prompt: A meditative companion robot inspired by serene Buddha-like aesthetics and contemplative wellness symbolism. Soft matte finish with warm, calming tones (mainly ivory, sand, muted gold, soft beige, and subtle wood accents). Smooth rounded head with closed or gently lowered eyes conveying peacefulness and mindfulness; minimal facial features with a subtle calm smile. Simplified organic body with harmonious proportions and flowing curved surfaces; minimal visible mechanical details. Gentle synchronized breathing movements with slow rhythmic upper-body motion; calm head inclinations and subtle meditative gestures. Minimalistic zen-inspired design language blending humanoid warmth with symbolic spiritual simplicity. Soft ambient lighting with warm diffuse glow; highly polished cinematic 3D rendering with smooth shadows and tranquil atmosphere.

- General: A meditative companion robot inspired by calmness, mindfulness, and contemplative wellbeing practices.
- Material & Color: Soft matte materials with calming natural tones (ivory, sand, muted gold, warm beige, subtle wood textures).
- Facial Features: Minimal serene face with softly closed or lowered eyes, subtle peaceful smile, rounded harmonious head shape.
- Body Details: Simplified organic humanoid form with flowing curves and balanced proportions; almost no exposed robotic mechanics.
- Movement & Interaction: Slow synchronized breathing motions; gentle head inclinations; calm and reassuring body language designed to encourage relaxation and emotional safety.
- Design Style: Minimalistic zen-inspired aesthetic combining symbolic spiritual design with soft humanoid embodiment; elegant, uncluttered, harmonious geometry.
- Effect / Impression: Peaceful, trustworthy, emotionally calming, reflective, and supportive; designed to evoke serenity, mindfulness, and emotional grounding.
- Rendering: Warm cinematic lighting; soft ambient glow; high-detail 3D rendering with smooth shadows, tranquil atmosphere, and premium wellness-product aesthetic.
