# Supplementary material for: Calm beats cute: designing aesthetic-functional congruence in human-robot interaction for breathing companion robots
Source: Front Cognit. 2026 Sep 23;5:1886795. doi: 10.3389/fcogn.2026.1886795 (PMC13642192; doi:10.3389/fcogn.2026.1886795)
Supplement: Supplementary file 3 [file Data_Sheet_3.pdf]

Calm Beats Cute: Designing Aesthetic-Functional Congruence in Human-Robot Interaction for Breathing Companion Robots.

Ilona Buchem[1], Rinat B. Rosenberg-Kima[2*], Emily Evermann[1] , Mandana Sutter[3]

[1]Laboratory or Communication, Department of Economics and Social Sciences, Berlin University of Applied Sciences, Berlin, Germany

[2]The Mindful Learning Technologies Lab, Faculty of Education in Science and Technology, Technion – Israel Institute of Technology, Haifa, Israel

[3]Institute of Psychology and Ergonomics, Faculty of Mechanical Engineering and Transport Systems Faculty, Technical University of Berlin, Berlin Germany

*** Correspondence:**
Ilona Buchem
buchem@bht-berlin.de

Keywords: aesthetics; social robots; human–robot interaction; embodiment; human-likeness; technology acceptance; user experience; breathing companion robot.

Supplementary Material 3

Table S3. Perceptions of Warmth, Competence, Enjoyment, and Intention to use by robot design.

**Measure**	**A**<br>**M (SD)**	**B**<br>**M (SD)**	**C**<br>**M (SD)**	**D**<br>**M (SD)**	**p**
**Warmth Total**	3.997 (0.800)	4.121 (0.734)	2.354 (1.036)	3.909 (0.846)	< .001
Friendly	4.311 (0.809)	4.330 (0.813)	2.151 (1.102)	3.915 (0.937)	< .001
Well-intentioned	4.000 (0.946)	4.198 (0.821)	2.717 (1.271)	4.057 (0.924)	< .001
Trustworthy	3.710 (0.915)	3.840 (0.937)	2.575 (1.203)	3.755 (1.012)	< .001
Warm	4.094 (0.961)	4.217 (0.884)	1.915 (1.172)	3.953 (0.960)	< .001
Good-natured	4.094 (0.921)	4.302 (0.818)	2.387 (1.151)	3.981 (0.976)	< .001
Sincere	3.774 (0.988)	3.840 (1.043)	2.377 (1.117)	3.792 (0.963)	< .001
**Competence Total**	3.453 (0.872)	3.432 (0.884)	3.281 (1.022)	3.753 (0.832)	< .001
Competent	3.528 (1.062)	3.500 (0.998)	3.377 (1.183)	3.811 (0.947)	.002
Confident	3.481 (1.026)	3.453 (1.043)	3.123 (1.102)	3.764 (0.962)	< .001
Capable	3.623 (0.961)	3.538 (1.053)	3.443 (1.130)	3.896 (0.915)	< .001
Efficient	3.566 (1.042)	3.575 (0.904)	3.491 (1.173)	3.745 (0.937)	.132
Intelligent	3.302 (1.071)	3.226 (1.124)	3.132 (1.180)	3.660 (1.022)	< .001
Skillful	3.217 (1.005)	3.302 (1.034)	3.123 (1.119)	3.642 (1.025)	< .001
**Perceived Enjoyment Total**	3.619 (1.071)	3.748 (1.057)	2.399 (1.161)	3.579 (1.033)	< .001

Enjoyable	3.613 (1.109)	3.736 (1.141)	2.434 (1.227)	3.613 (1.092)	< .001
Pleasant	3.774 (1.063)	3.858 (0.990)	2.462 (1.181)	3.783 (1.042)	< .001
Fun	3.472 (1.251)	3.651 (1.250)	2.302 (1.236)	3.340 (1.186)	< .001
**Behavioral Intention Total**	3.182 (1.263)	3.261 (1.250)	2.308 (1.143)	3.245 (1.246)	< .001
Use Intention	3.406 (1.217)	3.509 (1.148)	2.557 (1.288)	3.481 (1.228)	< .001
Acquisition Intention	3.094 (1.438)	3.028 (1.451)	2.104 (1.179)	3.047 (1.383)	< .001
Continued Use Intention	3.047 (1.355)	3.245 (1.344)	2.264 (1.237)	3.208 (1.293)	< .001
